# Supplementary material for: Child outcomes after induction of labour or expectant management in women with preterm prelabour rupture of membranes between 34 and 37 weeks of gestation: study protocol of the PPROMEXIL Follow-up trial. A long-term follow-up study of the randomised controlled trials PPROMEXIL and PPROMEXIL-2
Source: BMJ Open. 2021 Jun 15;11(6):e046046. doi: 10.1136/bmjopen-2020-046046 (PMC8208011; doi:10.1136/bmjopen-2020-046046)
Supplement: Supplementary data [file bmjopen-2020-046046supp002.pdf]

Additional file 2. SPIRIT schematic diagram of schedule of enrolment, interventions, and assessments of the women participating in PPROMEXIL trials and children participating in PPROMEXIL follow-up

	**STUDY PERIOD**<br>**Original PPROMEXIL trials - women**			**STUDY PERIOD**<br>**PPROMEXIL Follow-up - children**		
	**Enrolment original trials**	**Allocation original trials**	**Outcomes original trials**	**Enrolment follow-up study**	**Assessment follow-up study**	**Close-out**
**TIMEPOINT**	*Women with PPROM between 34 and 36+6 weeks of gestation*	t = 0	*Pregnancy, childbirth and neonatal period*	*After 10 – 12 years*	*Age 10-12 years*	*Age 10-12 years*
**ENROLMENT:**						
**Eligibility screen**	X			X		
**Informed consent**	X			X	X	
**Allocation**		X				
**INTERVENTIONS:**						
**Induction of Labor**		X				
**Expectant Management**		X				
**ASSESSMENTS:**						
Baseline variables	X	X		X		
Outcome variables			X		X	X
