# Supplementary material for: Child outcomes after induction of labour or expectant management in women with preterm prelabour rupture of membranes between 34 and 37 weeks of gestation: study protocol of the PPROMEXIL Follow-up trial. A long-term follow-up study of the randomised controlled trials PPROMEXIL and PPROMEXIL-2
Source: BMJ Open. 2021 Jun 15;11(6):e046046. doi: 10.1136/bmjopen-2020-046046 (PMC8208011; doi:10.1136/bmjopen-2020-046046)
Supplement: Supplementary data [file bmjopen-2020-046046supp003.pdf]

Additional file 3. GRIPP2 short form

Section and topic	Item	Reported on page No
1: Aim	Report the aim of PPI in the study	8, 20-21
2: Methods	Provide a clear description of the methods used for PPI in the study	8, 20-21
3: Study results	Outcomes—Report the results of PPI in the study, including both positive and negative outcomes	8
4: Discussion and conclusions	Outcomes—Comment on the extent to which PPI influenced the study overall. Describe positive and negative effects	NA
5: Reflections/critical perspective	Comment critically on the study, reflecting on the things that went well and those that did not, so others can learn from this experience	NA
