# Supplementary material for: Child outcomes after induction of labour or expectant management in women with preterm prelabour rupture of membranes between 34 and 37 weeks of gestation: study protocol of the PPROMEXIL Follow-up trial. A long-term follow-up study of the randomised controlled trials PPROMEXIL and PPROMEXIL-2
Source: BMJ Open. 2021 Jun 15;11(6):e046046. doi: 10.1136/bmjopen-2020-046046 (PMC8208011; doi:10.1136/bmjopen-2020-046046)
Supplement: Supplementary data [file bmjopen-2020-046046supp004.pdf]

Additional file 4. Patient information PPROMEXIL Follow-up (English version)

Patient information
PPROMEXIL Follow-up trial

Child outcomes after induction of labour or expectant management in women with preterm prelabour rupture of membranes between 34-37 weeks of gestation: the PPROMEXIL Follow-up trial, a long-term follow-up study of the randomised controlled trials PPROMEXIL and PPROMEXIL-2.

Dear Sir/Madame,

We would like to inform you on this research project called: 'Child outcomes after induction of labour or expectant management in women with preterm prelabour rupture of membranes between 34-37 weeks of gestation: the PPROMEXIL Follow-up trial, a long-term follow-up study of the randomised controlled trials PPROMEXIL and PPROMEXIL-2.'. You and your child have been asked to to take part in a medical-scientific study. Participation requires your written consent. In this letter we would like to inform you on the purpose of this research project and any advantages or disadvantages that it may hold for you. Please read this information carefully and do not hesitate to ask the investigator for an explanation if you have any questions. You can also ask the independent expert, who is mentioned at the end of this document, for additional information regarding the study protocol (page 4). And you may also discuss it with your partner, friends or family. Additional (general) information about participating in a study can be found in the enclosed general brochure on medical research.

Introduction
In the past you have participated in a trial called: PPROMEXIL or PPROMEXIL-2. You have participated in this trial because during your (last) pregnancy you have been diagnosed with premature preterm rupture of the fetal membranes (PPROM) at 34-37 weeks' gestational age. You have been treated with either expectant management or induction of labor. Short-term outcomes of your pregnancy and your child have been assessed at that time. In the proposed follow-up trial we would like to investigate offspring's long-term outcomes of women who participated in the PPROMEXIL trial; such as offspring's cognitive- and neurodevelopment, motor skills, behavioral development and general health.

Purpose of the research protocol
The goal of this study is to assess the long-term effects on children born to mothers whose pregnancy was complicated by PPROM between 34-37 weeks and who were treated with either induction of labor or expected management (PPROMEXIL and PPROMEXIL-2 trial). We would like to investigate child's cognitive development (intelligence), neurodevelopment

de Ruigh AA, *et al. BMJ Open* 2021; 11:e046046. doi: 10.1136/bmjopen-2020-046046

(fine and gross motor skills), academic attainment (school results), behavioral development and general health (diseases, hospital admissions, respiratory problems, but also length, weight, growth).

What participation involves

If you decide to participate in our research protocol we will contact you by phone. All data will be collected during one visit in a local or academic hospital in the neighborhood. During this visit children will be assessed on cognitive- and neurodevelopment and general health. Also physical examination will be obtained. Assessment of children has a playful approach, is enjoyable for most children and is not invasive. Furthermore, we will ask you to fill out questionnaires. Filling out these questionnaires will cost approximately 35 minutes. If necessary we would like to ask your permission to look up details on your (last) pregnancy and delivery in your medical chart. Furthermore, we would like to ask permission to obtain data from your child's consultation bureau or general practitioner.

Also, we would like to assess your child's academic attainment and school performance. Therefore we would like to ask the teacher at school to fill out a short questionnaire. We will ask the teacher whether your child needs any special education or additional support teaching.

If appreciated you can receive a short report on your child's cognitive, motor and behaviour development as measured by the different neurodevelopmental tests (WISC-V-NL, M-ABC-2, and CBCL). This short report will give information on total test scores and will tell you whether the test results of your child are above, on or below average. If the test results are below average, we will be advise you to contact your general practioner or a psychologist (in Dutch: 'GZ psycholoog of klinisch praktiserend psycholoog') for further help and interpretation of the different test scores.

de Ruigh AA, *et al. BMJ Open* 2021; 11:e046046. doi: 10.1136/bmjopen-2020-046046

Summary of study protocol

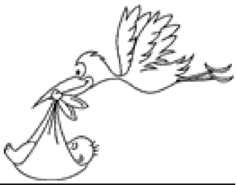

During your (last) pregnancy you have participated in the PPROMEXIL trial

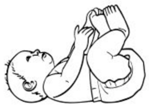

Short term outcomes of your child have been assessed shortly after birth

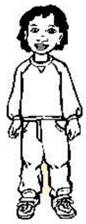

Years passed by and we would like to know how your son or daughter is developing. How is he/she doing at school? And is he/she good at sports?

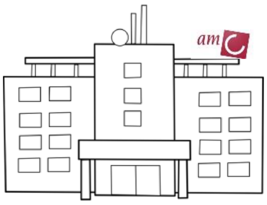

We would like to investigate your son or daughter in a local hospital in your neighborhood during a single visit

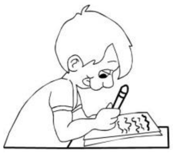

Together we will make some tests. Some of these tests may be easy, while others could be a little bit more difficult

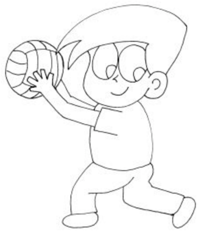

Also, we would like to investigate whether he or she is good at sports. And a physical examination will be obtained.

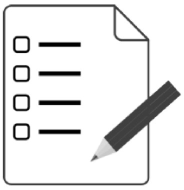

We would like to ask you to fill out four questionnaires about your son or daughter

de Ruigh AA, *et al. BMJ Open* 2021; 11:e046046. doi: 10.1136/bmjopen-2020-046046

Possible advantages and disadvantages
If you and your child participate in this research project it will cost time. Assessment of children has a playful approach, is enjoyable for most children and is not invasive. Participation in this trial is not associated with any risks.

You will not experience any personal benefit from participation in this study. However, your participation may contribute to more knowledge on the long-term (treatment) effects of induction of labour or expectant management on women's offspring after preterm prelabor rupture of the fetal membranes (PPROM) at a gestational age of 34-37 weeks.

Voluntary participation
It is up to you to decide whether or not you and your child will participate in the study. Participation is voluntary. If you do participate in the study, you can always change your mind and decide to stop, at any time during this study, without stating a reason. The data collected until that time will still be used for the study.
Your participation in this research will not change any decision making and quality of care that would be normally given to you or your child.

Confidential information
We assure that all data collected during the study will remain confidential. Data will be obtained in a coded manner. The investigator is the only person who will know which code you have. The key to the code will stay with the investigator. In the reports about the study only use this code will be used.You will not find your name in scientific papers.

Some people may access your medical and personal data. This is to check whether the study has been conducted in a good and reliable manner. General information about this policy can be found in the general brochure on medical research. People who may access your and your child's data are: the study team, a monitor of the study and the Healthcare Inspectorate. They will keep the data a secret. If you sign the consent form, you consent to your medical and personal data being collected, stored and accessed. The investigator will store your data for 15 years.

Study subject insurance
This study is not associated with any risks for you or your child. Therefore, the Academic Medical Center (AMC) does not need to take out additional insurance.

Compensation for participation
You and your child will not be paid for your participation in this study. You will be reimbursed for your travel costs.

Signing the consent form
When you have had sufficient time for reflection, you will be asked to decide on participation in this study. If you give permission, we will ask you to confirm this in writing on the appended consent form. By your written permission you indicate that you have understood the information and consent to participation in the study. The signature sheet is kept by your attending physician. You will get a copy or a second copy of this consent form.

de Ruigh AA, *et al. BMJ Open* 2021; 11:e046046. doi: 10.1136/bmjopen-2020-046046

Finally
If you have any questions, please contact the research team. You can contact one of the investigators (Noor Simons, PhD student, number; 020 – 5661470) or with the principal investigator in the AMC (Prof. dr. E. Pajkrt, gynecologist-perinatologist, number: 020 – 5661279). If you would like any independent advice about participation in this study, you may contact Dr. J.W. Ganzevoort, gynaecologist. He knows about the study but is not involved in it. Contact details: Dr. J.W. Ganzevoort, gynaecologist, department of Obstetrics and Gynaecology, Academic Medical Center (tel: 020-56 63769).

Thank you for your attention.

Kind Regards,

Prof. dr. E. Pajkrt,
Principal investigator
Professor Fetal and Maternal Medicine
Department of Gynecology and Obstetrics
Academic Medical Center, Amsterdam

Prof. dr. T. Roseboom
Professor of Early Development and Health
Department of Clinical Epidemiology, Biostatistics and Bioinformatics
Department of Gynecology and Obstetrics
Academic Medical Center, Amsterdam

de Ruigh AA, *et al. BMJ Open* 2021; 11:e046046. doi: 10.1136/bmjopen-2020-046046

Informed consent PPROMEXIL Follow-up - parents

- ✓ I have read the information sheet. I was also able to ask questions. My questions have been answered to my satisfaction. I have had enough time to decide whether me and my child will participate in this study.
- ✓ I know that participation is voluntary. I know that me and my child may decide at any time not to participate after all or to withdraw from the study.
- ✓ I know that some people will be able to access this person's personal data. These people are listed in this information sheet.
- ✓ I give permission for information to be requested from my gynaecologist about my pregnancy and delivery.
- ✓ I give permission to fill out questionnaires about my child, and I agree with one physical exam and one neurodevelopmental exam with my child (WISC-V and M-ABC-2).
- ✓ I give permission for information (regarding myself or my child) to be requested from my general practitioner or the GGD ('consultatiebureau').
- ✓ I know that it's possible to be contacted in the future (by post or telephone) after this study for another follow-up research project, if I give consent for this.
- ✓ I consent to my data and data regarding my child being stored at the research location for another 15 years after this study.

I agree to participation in this study

Date:____ - ____ - _________

First and last name child:: ……………………………………………………………………………………….

Child's date of birth: ……………………………………………………………………………………

First and last name child's parent/guardian: ………………………………………………………...

Signature child's parent/guardian:

First and last name child's second parent/guardian: ……………………………………………………

☐ Not applicable, because:……………………………………………………………….....

Signature child's second parent/guardian:

de Ruigh AA, *et al. BMJ Open* 2021; 11:e046046. doi: 10.1136/bmjopen-2020-046046

Additional:

I give consent for being contacted again (by post or by telephone) after this study for a follow-up study

☐ I do ☐ I do not

To be signed by the AMC investigator:

I declare that I have fully informed this/these person(s) about this study.

Name of investigator (or his/her representative):……………………………………………………………

Date:____ - ____ - _________

Signature:

The study subject will receive the full information sheet, together with a copy of the signed consent form.

de Ruigh AA, *et al. BMJ Open* 2021; 11:e046046. doi: 10.1136/bmjopen-2020-046046

Informed consent PPROMEXIL Follow-up – child 12 year and older

- I have read the information sheet. I was also able to ask questions. My questions have been answered to my satisfaction. I have had enough time to decide whether I will participate in this study.
- I know that participation is voluntary. I know that me and my child may decide at any time not to participate after all or to withdraw from the study.
- I know that some people will be able to access this person's personal data. These people are listed in this information sheet.
- I give permission to fill out one questionnaire, and I agree with one physical exam and one neurodevelopmental exam (WISC-V and M-ABC-2).
- I know that it's possible to be contacted in the future (by post or telephone) after this study for another follow-up research project, if I give consent for this.
- I consent to my data being stored at the research location for another 15 years after this study.

I have read all above and the information letter and want to participate with this research.

Date:____ - ____ - _________

First and last name child:………………………………………………………………………………………

Date of birth child: ………………………………………………………………………………………

Signature child: ………………………………………………………………………………………

My parent(s)/guardian(s) also agree with participation in this study and sign informed consent on a separate form.

de Ruigh AA, *et al. BMJ Open* 2021; 11:e046046. doi: 10.1136/bmjopen-2020-046046
