# Supplementary figures and images for: Child outcomes after induction of labour or expectant management in women with preterm prelabour rupture of membranes between 34 and 37 weeks of gestation: study protocol of the PPROMEXIL Follow-up trial. A long-term follow-up study of the randomised controlled trials PPROMEXIL and PPROMEXIL-2
Source: BMJ Open. 2021 Jun 15;11(6):e046046. doi: 10.1136/bmjopen-2020-046046 (PMC8208011; doi:10.1136/bmjopen-2020-046046)

**Additional file 5.** Direct Acyclic Graph (DAG) identifying potential confounding measures

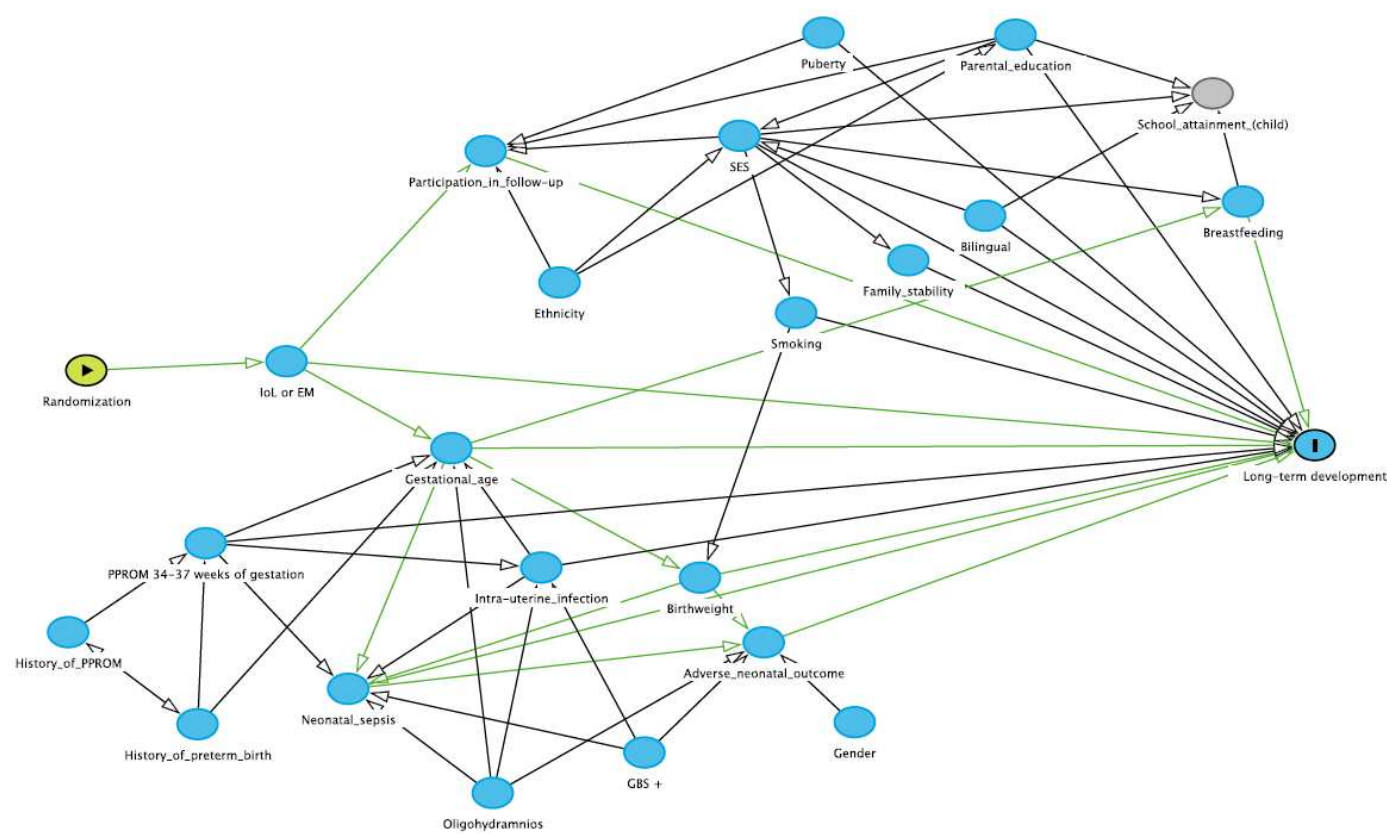

Supplement: Supplementary data [file bmjopen-2020-046046supp005.pdf]
