# Supplementary material for: Child outcomes after induction of labour or expectant management in women with preterm prelabour rupture of membranes between 34 and 37 weeks of gestation: study protocol of the PPROMEXIL Follow-up trial. A long-term follow-up study of the randomised controlled trials PPROMEXIL and PPROMEXIL-2
Source: BMJ Open. 2021 Jun 15;11(6):e046046. doi: 10.1136/bmjopen-2020-046046 (PMC8208011; doi:10.1136/bmjopen-2020-046046)
Supplement: Supplementary data [file bmjopen-2020-046046supp006.pdf]

Additional file 6.

Contributors to the PPROMEXIL and PPROMEXIL-2 trials

David P. van der Ham, Christine Willekes, Jantien L. van der Heyden, Sylvia M. C. Vijgen, Jan G. Nijhuis, Johannes J. van Beek, Brent C. Opmeer, Antonius L. M. Mulder, Rob Moonen, Mariët Groenewout, Mariëlle G. van Pampus, Gerald D. Mantel, Kitty W. M. Bloemenkamp, Wim J. van Wijngaarden, Marko Sikkema, Monique C. Haak, Paula J. M. Pernet, Martina Porath, Jan F. M. Molkenboer, Simone Kuppens, Anneke Kwee, Michael E. Kars, Mallory Woiski, Martin J. N. Weinans, Hajo I. J. Wildschut, Bettina M. C. Akerboom, Maureen T.M. Franssen, Christianne J.M. de Groot, J. Hans J. Duvekot, Bettina M.C. Akerboom, Aren J. van Loon, Jan W. de Leeuw, Ben Willem Mol, Aleid G. Leemhuis, E. Pajkrt, Martijn A. Oudijk, Bas Nij Bijvank, Caroline J. Bax, Janneke van ‘t Hooft.
