# Supplementary material for: Child outcomes after induction of labour or expectant management in women with preterm prelabour rupture of membranes between 34 and 37 weeks of gestation: study protocol of the PPROMEXIL Follow-up trial. A long-term follow-up study of the randomised controlled trials PPROMEXIL and PPROMEXIL-2
Source: BMJ Open. 2021 Jun 15;11(6):e046046. doi: 10.1136/bmjopen-2020-046046 (PMC8208011; doi:10.1136/bmjopen-2020-046046)
Supplement: Author's manuscript [file bmjopen-2020-046046.draft_revisions.pdf]

BMJ Open is committed to open peer review. As part of this commitment we make the peer review history of every article we publish publicly available.

When an article is published we post the peer reviewers' comments and the authors' responses online. We also post the versions of the paper that were used during peer review. These are the versions that the peer review comments apply to.

The versions of the paper that follow are the versions that were submitted during the peer review process. They are not the versions of record or the final published versions. They should not be cited or distributed as the published version of this manuscript.

BMJ Open is an open access journal and the full, final, typeset and author-corrected version of record of the manuscript is available on our site with no access controls, subscription charges or pay-per-view fees (http://bmjopen.bmj.com).

If you have any questions on BMJ Open's open peer review process please email info.bmjopen@bmj.com

BMJ Open

Child outcomes after induction of labour or expectant management in women with preterm prelabour rupture of membranes between 34-37 weeks of gestation: the PPROMEXIL Follow-up trial, a long-term follow-up study of the randomised controlled trials PPROMEXIL and PPROMEXIL-2.

Journal:	*BMJ Open*
Manuscript ID	bmjopen-2020-046046
Article Type:	Protocol
Date Submitted by the Author:	19-Oct-2020

Complete List of Authors:	de Ruigh, Annemijn; Amsterdam UMC Location AMC, Obstetrics and Gynaecology<br>Simons, Noor; Amsterdam UMC Location AMC, Obstetrics and Gynaecology<br>Van 't Hooft, Janneke; Amsterdam UMC Location AMC, Obstetrics and Gynaecology<br>van Wassenaer-Leemhuis, Aleid; Amsterdam UMC Location AMC, Department of Neonatology and Paediatrics, Emma Children's Hospital<br>Aarnoudse-Moens, Cornelieke; Amsterdam UMC Location AMC, Department of Neonatology and Paediatrics, Emma Children's Hospital<br>van Wely, Madelon; Amsterdam University Medical Centres, Netherlands Satellite of the Cochrane Gynaecology and Fertility Group<br>van Baaren, Gert-Jan; Amsterdam UMC Location AMC, Obstetrics and Gynaecology<br>Vlemmix, Floortje; Amsterdam UMC Location AMC, Obstetrics and Gynaecology<br>van der Ham, D.P.; Department of Obstetrics and Gynecology, Martini Hospital Groningen,<br>van Teeffelen, Augustinus; Maastricht UMC+, Obstetrics and Gynaecology<br>Mol, Ben; School of Medicine, Monash University, Melbourne, Australia, OB/GYN<br>Roseboom, Tessa; Amsterdam UMC Location AMC, Obstetrics and Gynaecology; Amsterdam UMC Locatie AMC, Department of Clinical Epidemiology, Biostatistics and Bioinformatics,<br>Pajkrt, Eva; Amsterdam UMC Location AMC, Obstetrics and Gynaecology

Keywords:	Fetal medicine < OBSTETRICS, Maternal medicine < OBSTETRICS, Prenatal diagnosis < OBSTETRICS, Developmental neurology & neurodisability < PAEDIATRICS

For peer review only - http://bmjopen.bmj.com/site/about/guidelines.xhtml

I, the Submitting Author has the right to grant and does grant on behalf of all authors of the Work (as defined in the below author licence), an exclusive licence and/or a non-exclusive licence for contributions from authors who are: i) UK Crown employees; ii) where BMJ has agreed a CC-BY licence shall apply, and/or iii) in accordance with the terms applicable for US Federal Government officers or employees acting as part of their official duties; on a worldwide, perpetual, irrevocable, royalty-free basis to BMJ Publishing Group Ltd ("BMJ") its licensees and where the relevant Journal is co-owned by BMJ to the co-owners of the Journal, to publish the Work in this journal and any other BMJ products and to exploit all rights, as set out in our licence.

The Submitting Author accepts and understands that any supply made under these terms is made by BMJ to the Submitting Author unless you are acting as an employee on behalf of your employer or a postgraduate student of an affiliated institution which is paying any applicable article publishing charge ("APC") for Open Access articles. Where the Submitting Author wishes to make the Work available on an Open Access basis (and intends to pay the relevant APC), the terms of reuse of such Open Access shall be governed by a Creative Commons licence – details of these licences and which Creative Commons licence will apply to this Work are set out in our licence referred to above.

Other than as permitted in any relevant BMJ Author's Self Archiving Policies, I confirm this Work has not been accepted for publication elsewhere, is not being considered for publication elsewhere and does not duplicate material already published. I confirm all authors consent to publication of this Work and authorise the granting of this licence.

For peer review only

Child outcomes after induction of labour or expectant management in women with preterm prelabour rupture of membranes between 34-37 weeks of gestation: the PPROMEXIL Follow-up trial, a long-term follow-up study of the randomised controlled trials PPROMEXIL and PPROMEXIL-2.

Annemijn A. de Ruigh, MD
Department of Obstetrics and Gynaecology, Academic Medical Centre (AMC), Amsterdam, The Netherlands

Noor E. Simons, MD
Department of Obstetrics and Gynaecology, Academic Medical Centre (AMC), Amsterdam, The Netherlands

Janneke van 't Hooft, MD PhD
Department of Obstetrics and Gynaecology, Academic Medical Centre (AMC), Amsterdam, The Netherlands

Aleid G. van Wassenaer-Leemhuis, MD PhD
Department of Neonatology and Paediatrics, Emma Children's Hospital, Academic Medical Center, Amsterdam, The Netherlands.

Cornelieke S.H. Aarnoudse-Moens, PhD
Department of Neonatology and Paediatrics, Emma Children's Hospital, Academic Medical Center, Amsterdam, The Netherlands.

For peer review only

Madelon van Wely, PhD
Centre for Reproductive Medicine, Academic Medical Center, Amsterdam, the Netherlands

Gert-Jan van Baaren MD PhD
Department of Obstetrics and Gynaecology, Academic Medical Center, Amsterdam, The Netherlands.

Floortje Vlemmix MD PhD
Department of Obstetrics and Gynaecology, Academic Medical Center, Amsterdam, The Netherlands.

David P van der Ham, MD, PhD
Department of Obstetrics and Gynaecology, Martini Hospital, Groningen, The Netherlands.

A.S.P. van Teeffelen, MD PhD
Department of Obstetrics and Gynaecology, GROW - School for Oncology and Developmental Biology, Maastricht University Medical Centre, Maastricht, The Netherlands.

Ben Willem Mol, MD PhD
Obstetrics and Gynaecology, Monash University, Melbourne, Victoria, Australia

Tessa J. Roseboom, PhD
Department of Clinical Epidemiology, Biostatistics and Bioinformatics, Academic Medical Centre, University of Amsterdam, Amsterdam, The Netherlands; Department of Obstetrics and Gynaecology, Academic Medical Centre, University of Amsterdam, The Netherlands.

Eva Pajkrt, MD, PhD

For peer review only

For peer review only - http://bmjopen.bmj.com/site/about/guidelines.xhtml

Department of Obstetrics and Gynaecology, Academic Medical Centre (AMC), Amsterdam, The Netherlands

Word count: Total word count main text: 4,273

Disclosure: Dr. Ben Willem Mol is supported by a NHMRC Practitioner Fellowship (GNT1082548). Dr. Ben Willem Mol reports consultancy for ObsEva, Merck Merck KGaA and Guerbet. All other authors did not report any conflicts of interest.

Financial Support: This study is funded by ZonMW (grant number 843002826).

Corresponding Author: Annemijn A. de Ruigh, MD, a.a.deruigh@amsterdamumc.nl
Department of Obstetrics and Gynaecology, Academic Medical Center (AMC), Meibergdreef 9, 1105, AZ, Amsterdam, The Netherlands

For peer review only

For peer review only - http://bmjopen.bmj.com/site/about/guidelines.xhtml

ABSTRACT

Introduction: Late preterm prelabour rupture of membranes (PROM between 34^{+0} and 36^{+6} weeks gestational age) is an important clinical dilemma. Previously, two large Dutch randomised controlled trials (RCTs) compared induction of labour to expectant management. Both trials showed that early delivery does not reduce the risk of neonatal sepsis as compared to expectant management, although prematurity related risks might increase. An extensive, structured long-term follow-up of these children has never been performed.

Methods and analysis: The PPROMEXIL Follow-up trial aims to assess long-term childhood outcomes of the PPROMEXIL (ISRCTN29313500) and PPROMEXIL-2 trial (MEC 05-240, ISRCTN05689407), two multicenter RCTs using the same protocol, conducted between 2007-2010 evaluating induction of labour versus expectant management in women with late preterm PROM. The PPROMEXIL Follow-up trial will analyse children of mothers with a singleton pregnancy (induction of labour n=359; expectant management n=353). At 10-12 years of (corrected) age all surviving children will be invited for a neurodevelopmental assessment using the Wechsler Intelligence Scale for Children-V, Color-Word Interference Test and the Movement Assessment Battery for Children-2. Parents will be asked to fill out questionnaires assessing behaviour, motor function, sensory processing, respiratory problems, general health and need for health care services. Teachers will fill out the Teacher Report Form and answer questions regarding school attainment. For all tests means with SD's will be compared, as well as predefined cut-off scores for abnormal outcome. Sensitivity analyses consisting of different imputation techniques will be used to deal with loss-to-follow-up.

Ethics and dissemination: The study has been granted approval by the MEC of the AmsterdamUMC (MEC2016_217). Results will be disseminated through peer-reviewed journals and summaries shared with stakeholders. This protocol is published before analysis of the results.

For peer review only - http://bmjopen.bmj.com/site/about/guidelines.xhtml

Registration: Dutch Trial registration number: NTR6953 (registration December 28th, 2017).

The study has been peer reviewed, approved and funded by ZonMW (843002826).

Key words: Late preterm prelabour rupture of membranes, induction of labour, expectant management, long-term outcome, child development, child health

For peer review only

For peer review only - http://bmjopen.bmj.com/site/about/guidelines.xhtml

ARTICLE SUMMARY

Strengths and limitations of this study

- This long-term follow-up study will be the first study to evaluate long-term developmental outcomes (cognitive, motor, and behavioural development, sensory processing, respiratory problems, general health, children's need for health-care services, and school attainment) in the offspring of women who have been treated during pregnancy with induction of labour or expectant management for late preterm prelabour rupture of membranes.
- Children will be evaluated at 10-12 years of age with internationally validated measurements and questionnaires, translated for Dutch children, using norm scores for Dutch children.
- A trained team consisting of a (neuro)psychologist and physician, masked to the study group, will perform all neurodevelopmental tests.
- The study will be performed within the Dutch Consortium for Healthcare Evaluation and Research in Obstetrics and Gynecology - NVOG Consortium 2.0, a collaboration of approximately 70 obstetric hospitals (academic and non-academic hospitals) in the Netherlands.
- Alongside this long-term follow-up study a separately reported economic evaluation study will be planned to investigate cost-effectiveness of both treatments taking long-term developmental outcomes into account.

For peer review only

INTRODUCTION

Background and rationale

Late preterm prelabour rupture of membranes (late preterm PROM) between 34^{+0} and 36^{+6} weeks gestation, is an important clinical problem occurring in 1.5% of pregnant women, of which 25% will deliver within 24 hours.(1) After PROM, the risk of infection increases for both mother and foetus. Recently, three large randomised controlled trials (RCTs) compared induction of labour to expectant management for women whose pregnancy was complicated by late preterm PROM.(2-4) The Dutch PPROMEXIL and PPROMEXIL-2 trial, and the Australian PPROMT trial showed that induction of labour does not reduce the risk of neonatal sepsis as compared to expectant management, while increasing prematurity related risks, such as hypoglycaemia and hyperbilirubinemia. Furthermore, an Individual Participant Data Meta-analysis (IPD-MA) investigating participant data of all three RCTs also concluded that expectant management is an acceptable alternative to induction of labour, as both treatments resulted in comparable rates of a composite of adverse neonatal outcomes.(5) Moreover, an economic analysis of the PPROMEXIL trial, showed that health care costs for induction of labour are slightly higher, although not statistically significant, with a mean difference of €754 (€8,094 for induction of labour versus €7,340 for expectant management, 95% confidence interval (CI) -€335 to €1,802).(6) Therefore, currently most national guidelines advocate expectant management for late preterm PROM.(1, 7, 8)

In 2015 our research team performed a follow-up study of children at two years of age, born to women who participated in the PPROMEXIL trial.(9) This follow-up study was performed with limited budget and used internationally validated screening questionnaires. Even though this study had a follow-up rate of 44% and no extensive neurodevelopmental assessments were used, an increase in neurodevelopmental impairment was found in the expectant management group as compared to the induction of labour group (abnormal score (-2 standard deviation (SD)) in ≥1 domains of the Ages and Stages Questionnaire: 14%

For peer review only - http://bmjopen.bmj.com/site/about/guidelines.xhtml

induction of labour group versus 26% expectant management group, difference in percentage -11.4; 95% CI -21.9 to -0.98).(9) Hypothetically, a prolonged stay of the foetus in an environment at risk for (subclinical) infections such as maternal placental inflammation (histological or clinical chorioamnionitis) and foetal side placental inflammation (funisitis and chorionic plate vasculitis) in case of expectant management could affect brain development (i.e. neurological outcome) and therefore explain the neurodevelopmental impairment seen at 2 years of age.(10) The developmental effects of induction of labour or expectant management after late preterm PROM in children after 2 years are still unknown. Furthermore, understanding the long-term effects on women's offspring of either treatment is important for both clinicians and pregnant women when deciding how to manage late preterm PROM.

Until now, no other study has performed or planned a comprehensive long-term follow-up of children born after late preterm PROM. Study feasibility was investigated by an online questionnaire filled out by parents and members of a Dutch patient organization representing patients affected by preterm birth due to complications in pregnancy. Results showed that 89% of parents were willing to participate in an extensive follow-up study. Parents rated the outcomes general health, behaviour, school attainment and respiratory problems as most important outcomes (data not published). Additionally, a focus group meeting with mothers of prematurely born children with or without preterm PROM was organized, to discuss the different aspects of the child's long-term development. In general, mothers expressed concerns regarding cognitive development, in particular executive functions, motor development, social interaction and behaviour and the level of independency the child will attain later in life (data not published).

Objectives

Therefore, the aim of this study is to conduct a structured follow-up of all children born to women with late preterm PROM who were randomised to induction of labour or expectant

For peer review only - http://bmjopen.bmj.com/site/about/guidelines.xhtml

180 management in the PPROMEXIL and PPROMEXL-2 trial. Long-term cognitive, motor, and behavioural development, sensory processing, respiratory problems, general health, children's need for health-care services, and school attainment will be assessed at 10-12 years of age using internationally validated measurements and questionnaires, translated and using norm scores for Dutch children.

For peer review only

METHODS AND ANALYSIS

Study setting

We will perform an extensive long-term follow-up study of two previously executed RCTs (PPROMEXIL Follow-up trial, NTR 6953, METC 2016_217, NL58494.018.16) investigating long-term developmental outcomes (cognitive, motor, behavioural development), sensory processing, respiratory problems, general health, children's need for health-care services, and school attainment. This will be assessed at 10-12 years of corrected age in children born to women with a singleton pregnancy complicated by late preterm PROM (between 34+0 and 36+6 weeks gestation), who participated in the RCTs PPROMEXIL, and PPROMEXIL-2 trial. Details of the PPROMEXIL (ISRCTN29313500) and PPROMEXIL-2 trial (amendment of the PPROMEXIL trial (MEC 05-240), ISRCTN05689407) have been published elsewhere.(2, 3) These two large RCTs, using the same study protocol and conducted between 2007 and 2011 in 61 academic and non-academic hospitals in The Netherlands, assessed whether induction of labour versus expectant management would reduce the incidence of neonatal sepsis in women with late preterm PROM. In the induction of labour group, patients were induced within 24 hours after randomization. Patients in the expectant management group were monitored until the onset of spontaneous delivery or induced after 37+0 weeks according to national guidelines.(1)

Participants and eligibility criteria

All children born to women with a singleton pregnancy who participated in the PPROMEXIL trials will be invited for this long-term follow-up assessment. Children will be evaluated at 10-12 years of age. As the total number of multiple pregnancies in the PPROMEXIL- and PPROMEXIL-2 trials was very low (14/727 (1.9%) and equally distributed among treatment groups), only singleton pregnancies will be included in the analysis. See Figure 1. for the overview of PPROMEXIL follow-up participants.

For peer review only

Procedures and recruitment

The study protocol is designed, constructed and reported according to the recommendations given in the Standard Protocol Items: Recommendations for Interventional Trials (See Additional file 1. SPIRIT checklist for reporting randomised trials; and Additional file 2. SPIRIT template for visualizing schedule of enrolment, interventions, and assessments of the women participating in PPROMEXIL trials and children participating in PPROMEXIL follow-up.)(11) The study will be performed within the Dutch Consortium for Healthcare Evaluation and Research in Obstetrics and Gynecology - NVOG Consortium 2.0, a collaboration of approximately 70 obstetric hospitals (academic and non-academic hospitals) in the Netherlands (https://zorgevaluatienederland.nl/nvog). Research nurses will be asked to crosscheck medical records for possible occurrence of death of women's offspring before contacting parents and their child for participating in this follow-up study. All parents will be contacted by post to announce this follow-up study, and if they give consent to be approached by the research team, they will be contacted by telephone or email to explain study details. Parents will be informed that participation is voluntary and that they may withdraw consent to participate at any time. They will be informed that declining participation will not affect their or their child's care. Parents will be given sufficient time to read the patient information and they will be given the opportunity to ask questions by telephone or email prior to signing the informed consent form. Written study information at children's reading level will be available for all children (specified for children <12 years of age and ≥12 years of age. An independent physician (i.e. not a member of the research team) will be available to answer any questions patients may have. Written informed consent will be obtained from both parents prior to the examination. Children ≥12 years of age have to sign their own informed consent, in addition to the informed consent of their parents, at the day of the assessment. A copy of the informed consent form(s) will be given to the parents/child. All study documents will be available through the study website.

For peer review only - http://bmjopen.bmj.com/site/about/guidelines.xhtml

Concealment of treatment allocation at time of the PPROMEXIL and PPROMEXIL-2 trials (i.e. induction of labour or expectant management) was not possible due to the type of intervention, and therefore parents and children entered in this follow-up study will be aware of treatment allocation. The research team performing the follow-up examinations and all members of the research team performing data entry and data analyses will be masked to treatment allocation.

All data will be collected, captured, and coded in accordance with existing polices to ensure patient confidentiality. Data will be recorded using an electronic case record form and will be stored in a web-secured database (available through the study website).(12) The investigators will publish the results of this trial in a peer reviewed medical journal as soon as appropriate. The Clinical Research Unit (CRU) of the Amsterdam UMC will monitor data collection.

Follow-up assessment and outcomes

During a single visit in an outpatient clinic of a local hospital close to the family's neighbourhood, children will be assessed on long-term neurodevelopmental outcomes using standardized and validated neurodevelopmental tests and questionnaires. A trained team consisting of a (neuro)psychologist and physician, masked to the study group, will perform all neurodevelopmental tests. Neurodevelopmental assessment of children has a structured approach, is enjoyable for most children and is not invasive. During neurodevelopmental assessment of the child, parents will be asked to fill out questionnaires on sensory processing, behaviour, respiratory problems, and child's health. If necessary, parents will be assisted with filling out questionnaires. All, but one, questionnaires are digital and can be filled out on a tablet during the assessment or at any other time at home.

After completing all examinations, parents receive a short report on their child's test results. This short report will give information on total test scores and tell parents whether their child's

For peer review only - http://bmjopen.bmj.com/site/about/guidelines.xhtml

scores are above, on or below average. If test scores indicate that children would benefit from supportive (health, developmental or educational) care, parents are advised to contact their general practitioner for referral to a paediatrician or psychologist.

We consider our main outcomes to be: cognitive development (assessed by the WISC-V), motor skills (assessed by the M-ABC-2) and behaviour (assessed by the CBCL).

Assessment of cognitive development

Cognitive Development will be assessment using the Dutch version of the Wechsler Intelligence Scale for Children (WISC-V).(13) The WISC-V is used worldwide to assess cognition in children aged six to 16 years and consists of 10 subtests that are combined into a Full Scale IQ score (FSIQ) and five primary indexes: verbal comprehension, visual spatial, fluid reasoning, working memory and processing speed. Besides these primary indexes, an additional mathematics subtest will be obtained to provide an objective measurement of this area of academic attainment. The WISC-V total intelligence quotient (IQ) score and primary indexes have a mean score of 100 points with a SD of 15 points. An index score ≤70 (≥ -2 SD below the mean score) will be considered as a severe cognitive delay and will be compared between groups. An index score >70 and ≤84 (≥ -1 SD and < -2SD below the mean score) will indicate a mild cognitive delay. Normal cognitive outcome is defined as no severe or mild neurodevelopmental delay. A difference between the two treatment groups of 7.5 points (0.5 SD) could indicate a potential clinical relevant difference.

Child's executive functioning will be tested using subtests of the WISC-V and the Color-Word Interference Test (CWIT). The CWIT measures cognitive set shifting and the ability to inhibit a dominant and automatic verbal response by separate and combined Color Naming and Color Reading items. The CWIT subtests have a mean of 10 points with a SD of 2 points. An CWIT index score of ≤4 (i.e. more than -2 SD below the mean score) is considered a severe delay in executive functioning and will be analysed.

For peer review only - http://bmjopen.bmj.com/site/about/guidelines.xhtml

Assessment of motor skills

Child's motor function will be measured by the Movement Assessment Battery for Children-2 (M-ABC-2).(14) The M-ABC-2 is the most commonly used tool used to examine fine and gross motor skills. The M-ABC-2 provides data about a child's performance of age-appropriate tasks within three domains; manual dexterity, aiming and catching, and balance. M-ABC-2 scores will be calculated as standard scores and percentiles for each domain, and as a total test score. The mean standard score for all domains and the total score is 10 points, with a SD of 3 points. The age band two (7-10 years of age) and three (11-16 years of age) of the M-ABC-2 will be used, as appropriate according to the child's age. A standard score of ≤5 points, representing ≤5th percentile will be defined as a significant movement difficulty and a severe delay in motor skills and will be compared between treatment groups. A standard score of 6 or 7 points, representing >5th to ≤16th percentile will indicate that the child is at risk of having a movement difficulty and therefore will be classified as mild delay in motor skills. A standard score of ≥8 points, representing>16th percentile will be defined as no movement difficulty and normal development of motor skills.

Furthermore, parents will fill out the Movement-ABC-2 checklist, a questionnaire that consists of three sections on movement in static and/or predictable environment, movement in a dynamic and/or unpredictable environment and non-motor factors that may affect the child's movement. The sections on static and dynamic movements are summed up to a total score, with a higher score indicating a worse motor function. A total score of ≥95th percentile (≥9 points) indicates severe motor impairment and will be compared between both treatment groups.

Assessment of behavioural development

Child's behaviour will be measured by the Child Behaviour Checklist (CBCL), a parental questionnaire used to screen for behaviour problems in children.(15) It informs on eight narrow syndrome scales (anxious/depressed, withdrawn/depressed, somatic complaints, social problems, thought problems, attention problems, rule-breaking behaviour, and

For peer review only - http://bmjopen.bmj.com/site/about/guidelines.xhtml

aggressive behavior) and three broadband scales (internalizing, externalizing behavioural problems and a total problems score) which are composed out of the narrow-band syndrome scales. The CBCL broadband scales T scores have a mean of 50 points with a SD of 10 points. A score >90th percentile (>63 points) on one of the two broad dimensions scales (internalizing problems or externalizing problems), or the total problem score (sum of all scores) of the CBCL will be defined as abnormal and clinically relevant for indicating behavioural problems. Scores ≥84st and ≤90th percentile (≥60 and ≤63 points) are considered borderline and scores <84th percentile (<60 points) are defined as normal.

Assessment of school attainment

Child's academic attainment and behaviour will be assessed using the Teacher's Report Form (TRF).(16) The TRF assesses problem behaviour in the last two months and identifies the same eight syndromes as the CBCL, and also inquires on academic attainment (Academic Performance). With parental permission, the TRF will be filled out by the child's school teacher (the teacher who has known the child in the school setting for more than two months can complete the TRF). Accompanying the TRF, teachers will be asked some additional questions regarding the child's need for additional educational support in- or outside the classroom. For the TRF the cut-off percentiles of the broad band and total scores as used in the CBCL will be applied. For Academic Performance a cut-off score of <10th percentile (≤36 points) will be defined as abnormal. Scores between 10th and 16th percentile are classified as borderline and ≥17th percentile are considered normal outcome.

Assessment of sensory processing

Sensory processing will be determined using the Short Sensory Profile questionnaire (SSP-NL).(17) The Short Sensory Profile contains sections corresponding to each sensory system, sections that indicate the modulation of sensory input across sensory systems, and sections that indicate behavioural and emotional responses that are associated with sensory processing. This questionnaire consists of 38 items, classified into seven subscales (Tactile Sensitivity, Taste/Smell Sensitivity, Movement Sensitivity, Underresponsive/Seeks

For peer review only - http://bmjopen.bmj.com/site/about/guidelines.xhtml

Sensation, Auditory Filtering, Low Energy/Weak, and Visual/Auditory Sensitivity). For every subscale parents will be asked how frequently their children respond in the way described by each item using a 5 point Likert scale (nearly never, seldom, occasionally, frequently, almost always). Lower scores on the total score and subscales indicate more sensory symptoms. Subscales and the total scores will be used to classify as “definite difference” (cut off scores ≥-2 SD below the mean) and will be compared between groups. “Typical performance” will be defined as < -1 SD below the mean, “probable difference” will be defined as ≥ -1 SD and < -2 SD below the mean.

Assessment of respiratory problems

Respiratory problems, such as asthma or other lung problems will be assessed using the International Study of Asthma and Allergies in Childhood questionnaire (ISAAC questionnaire) which informs on asthma, rhinitis and eczema.(18) The diagnosis of asthma will be defined as a positive answers to the question: “In the last 12 (twelve) months, has your child had wheezing?”, as this question has a sensitivity of 100%, specificity of 78%, positive predictive value of 73%, and negative predictive value of 100% for the diagnosis of asthma.(19)

Assessment of anthropometry and pubertal status

Children will be asked to fill out the Puberty Developmental Scale (PDS), a self-report measure of pubertal status.(20) Children will be asked questions regarding on e.g. growth in height, skin changes, body or facial hair, deepening of the voice (for boys), and starting to menstruate or developing breasts (for girls). Physical examination will be restricted to measurement of height/weight and blood pressure. Results of physical examination (height/weight, body mass index) will be used for baseline characteristics. Puberty status will be used for baseline characteristics and subgroup analysis.

Assessment of child’s health and need for health care services

A general questionnaire consisting of 61 items, will be used to assess demographic

For peer review only - http://bmjopen.bmj.com/site/about/guidelines.xhtml

characteristics and will ask questions regarding the present (last 12 months) and the past health and health care use (from discharge after delivery until date of assessment) (also used in previous follow-up studies such as ProTWIN*kids* study at three and four years, TripleP study(21-23)). Questions address child's health, need for health care services, hospital visits, hospital submission, need for surgery, use of medication, psychological problems, need for developmental therapies (such as physical therapists, remedial teaching, speech therapist, occupational therapist). Health care use and (health) related problems will be clustered into different clinically relevant groups (e.g. need for medical specialist and/or developmental care, medication use in the past and present, hospital admissions and surgery to give insight in the range of health related problems).

Parents will be asked to give permission to gather medical information on the child's health via the general practitioner and the preventive youth healthcare services if needed.

Economic analysis

Alongside this long-term follow-up study, an economic evaluation study will be planned to investigate cost-effectiveness of both treatments taking long-term developmental outcomes into account. Results of this economic evaluation will be reported separately from trial results.

At present, no additional long-term follow-up in later life (>12 years of age) is planned. Permission to approach parents and children for additional follow-up research in later life will be obtained with informed consent form during the current follow-up study. If additional long-term follow-up of children at an adolescence age will be planned in the future, additional approval of the Medical Research Ethics Committee will be sought.

Sample size

Since this is a follow up study, the maximum number of study participants is already defined by the two PPROMEXIL trials, excluding multiple pregnancies and deceased children (Figure

For peer review only

For peer review only - http://bmjopen.bmj.com/site/about/guidelines.xhtml

1 and Additional file 2.). Consequently, 712 children are eligible for inclusion, 359 born in the induction of labour group and 353 born in the expectant management group. As we will not be able to adjust the number of recruited children, a power calculation will not be of any use to calculate a study sample size. However, this calculation can indicate the minimum number of children that need to be tested in order to find a clinically significant difference for the three most important outcomes in this study: cognitive development, motor skills and behavioural development. All sample size calculations are with a power of 90%, a two-sided a of 0.05 and ß of 0.20. To be able to detect a clinically relevant difference in mean scores of 0.5 SD in all tests, 86 children per group will be sufficient (total 172 children). This 0.5 SD equals a difference of 7.5 IQ points in the mean score of the WISC-V test (cognitive development), a difference of 1.5 points on the mean total standard scores of the M-ABC-2 (motor skills) and a difference of 5 points on the mean T scores in any of the broadband problem scales of the CBCL (behavioral development) between both groups. Thus, since 172 children comprise 24% of our total, also in case of limited follow up, differences of 0.5 SD can be picked up. Based on previous experience in our research team with follow-up trials and based of existing literature, we expect to have a follow-up rate of 30 to 40% of the children.(25)

Statistical methods

Differences in background characteristics and the maternal, pregnancy, delivery and neonatal outcomes between the induction of labour group and expectant management group will be compared using unpaired T-test, Mann-Whitney U test, Chi-square test or Fisher's exact test when appropriate. The same characteristics will be compared in children assessed at follow-up and for the original participants of the PPROMEXIL trials. This will allow us to assess whether selection or attrition bias may be present in our study (e.g. due to drop-out of healthy or unhealthy children). To compare the long-term developmental outcomes between both treatment groups mean differences and the corresponding 95% CI will be calculated. For dichotomous outcomes relative risk (RR) with corresponding 95% CI will be calculated.

For peer review only - http://bmjopen.bmj.com/site/about/guidelines.xhtml

Our main analyses shall be based upon the results from the children assessed in follow-up (complete case analysis).(24)

The relatively simple statistical analysis described above can be justified by the fact that our study is a follow-up of two RCTs and consequently no confounding measures are expectant (See Additional file 4. Direct Acyclic Graph (DAG)). The DAG confirms that there are no variables susceptive to have influenced the likelihood of receiving the intervention and subsequently have influenced long-term outcome of the child. On the other hand, selection bias may occur as a consequence of incomplete follow-up. We will evaluate the effect of differences in background characteristics (such as maternal smoking, social-economic status) and if applicable, report unadjusted and adjusted odds ratios for dichotomous outcomes using logistic models and adjusted mean differences and the corresponding 95% CI for continuous outcomes using general linear models.

Sensitivity analyses

Our pre-planned sensitivity analyses will only be performed for the WISC-V, the Movement-ABC and the CBCL total scores to minimize the effect of multiple testing.

Imputation missing data: A sensitivity analysis using imputation techniques will be performed to impute missing data for children that are lost to follow-up. Imputation techniques will only be applied when it can be assumed that data is (mostly) missing at random and the follow-up rate is follow-up rate ≥70% (the group agreed on an arbitrary). If the loss to follow-up rate is higher a best- and worst-case scenario will be performed. In these two scenarios the missing cases are imputed either all as 'normal' (best case) or as 'abnormal' (worst case) outcomes. These scenarios will provide some insight on the robustness of the complete case follow-up results.

Age and puberty adjusted scores: Despite the fact that most children are born late premature/near term or full term, a sensitivity analyses will be performed using age-adjusted scores (corrected for prematurity). Finally, a sensitivity analysis using results of the PDS

For peer review only

indicating child's puberty status will be performed.

Subgroup analyses exploring the potential impact of effect modification

Dealing with the effect of 'down-stream' factors: During time to follow-up (due to loss to follow-up) a substantial difference in the prevalence 'down-stream' factors could potentially appear ('down-stream' factors are defined as potential effect modifiers appearing after randomization, such as sepsis at birth, positive GBS). In sensitivity analysis the potential interaction of the following 'down-stream' factors will be explored: gestational age at PROM, receiving antibiotics, receiving steroids for fetal maturation, receiving tocolysis, group B streptococci (GBS) positivity, a positive vaginal culture (including GBS and other pathogens not consistent with normal flora), neonatal sepsis, and for women who participated in the former follow-up study of children at 2 years of age.(9) The analysis will be stratified by these different factors and the potential differences in long term outcomes between the different strata will be explored.

A P-value of <0.05 was considered to indicate statistical significance. All analyses will be performed according to the intention-to-treat principle using IBM SPSS (NY, USA) or in RStudio (Boston, MA).

A statistical analysis plan (SAP), reporting a more detailed description of the statistical methods and analyses, will be published separately from the PPROMEXIL follow-up protocol.

Patient and public involvement

The Dutch association for parents of incubator children and the Dutch Collaboration of parent- and patient organisations endorsed the study and provided input on the study proposal. Parents from the Dutch association for parents of incubator children participated in an online survey. Additionally, mothers of prematurely born children participated in a focus

For peer review only - http://bmjopen.bmj.com/site/about/guidelines.xhtml

group meeting organized by our research team, to discuss the different aspects of child's long-term development to incorporate in long-term follow-up research.

DISCUSSION

Long-term follow-up of all children born to mothers participating in obstetric intervention trials is of crucial importance.(25) The outcome late neurodevelopmental morbidity has been identified and selected by parents as one of 13 core outcomes for studies evaluating preventive interventions for preterm birth.(26) Furthermore, previous studies have stressed the importance of long-term follow-up by demonstrating that interventions performed during pregnancy can have unexpected long-term effects on children which may not be apparent at birth or during neonatal assessment.(27) By assessing cognition, motor function, behaviour, respiratory problems, general health and school attainment in an extensive and structured follow-up, this study will have the unique opportunity to help understanding the long-term effects of our current treatment regimen for late preterm PROM on women's offspring.

For peer review only

ETHICS AND DISSEMINATION

Ethics approval and consent to participate

The PPROMEXIL Follow-up trial aims to assess long-term childhood outcomes of the PPROMEXIL trial (ISRCTN29313500) and PPROMEXIL-2 trial (MEC 05-240, ISRCTN05689407), two multicentre RCTs using the same study protocol. The Medical Ethics Committee of the Academic Medical Centre Amsterdam (MEC) has approved the PPROMEXIL Follow-up trial (MEC2016_217, NL58494.018.16). Table 1 describes the chronology of submission and amendments to the MEC.

For peer review only

Table 1.

Chronology submission and revisions PPROMEXIL follow-up study

**Version** no.	**Date** (DD-MM-YYYY)	**Main reasons for change**
**1**	29-09-2016	N/A, first submission to MEC
**2**	20-12-2017	MEC2016_217#C20161752<br>Modifications requested by MEC d.d. 05-09-2016
**3**	04-01-2018	Modifications requested by MEC d.d. 04-01-2018<br>Additional information on informed consent about saving data (mother and child) up to 15 years after trial
**3**	10-01-2018	Approval MEC d.d. 10-01-2018
**4**	31-05-2018	Amendment 1:<br>- Administrative modifications<br>- Change of acronym to PPROMEXIL follow-up<br>- Addition of two questionnaires (ISAAC and Puberty developmental scale)
**4**	04-07-2018	Approval amendment version 4 d.d. 04-07-2018
**5**	10-08-2018	Amendment 2:<br>- Modification in protocol on how to recruit participants

		(via research nurses and PhD student)<br>- Modification in Patient Information Files on recruitment
**5**	23-08-2018	Approval amendment version 5 d.d. 23-08-2018
**6**	06-11-2018	Amendment 3:<br>- Clarification on informed consent procedure, parents and participants will be counseled through the telephone and sign informed consent at home<br>- Minor modifications in the general health questionnaire
**6**	23-11-2018	Approval amendment version 6 d.d. 23-11-2018
**7**	03-04-2019	Amendment 4:<br>- Addition of patient information files for children age 12-15
**7**	12-04-2019	Approval amendment version 7 d.d. 12-04-2019

See https://www.zorgevaluatienederland.nl/evaluations/ppromexil-follow-up for the full study protocol and electronic case record form. Written informed consent will be obtained from both parents prior to the examination. Children ≥12 years of age have to sign their own informed consent, in addition to the informed consent of their parents, at the day of the assessment. A copy of the informed consent form(s) will be given to the parents/child.

Dissemination

No arrangements have been made concerning public disclosure. The trial is registered in the Dutch Trial register (Trial registration number: NTR6953. Date of registration December 28th 2017). An overview of the WHO trial registration data set is described in Table 2.

For peer review only - http://bmjopen.bmj.com/site/about/guidelines.xhtml

524 **Table 2.**

525 **WHO trial registration data set**

Primary Registry and Trial Identifying Number	Trial NL6623 (NTR6953)
Date of Registration in Primary Registry	December 28th, 2017
Secondary Identifying Numbers	n/a
Source(s) of Monetary or Material Support	ZonMW Dutch Healthcare efficacy program
Primary Sponsor	Academical Medical Center (AMC), Amsterdam, The Netherland
Secondary Sponsor(s)	n/a
Contact for Public Queries	Drs. Noor Simons<br>Followup.ppromexil@amsterdamumc.nl
Contact for Scientific Queries	Prof. dr. Eva Pajkrt<br>e.pajkrt@amsterdamumc.nl
Public Title	PPROMEXIL follow-up
Scientific Title	Child outcomes after induction of labour or expectant management in women with preterm prelabour rupture of membranes between 34-37 weeks of gestation: the PPROMEXIL Follow-up trial, a long-term follow-up study of the randomised controlled trials PPROMEXIL and PPROMEXIL-2.
Countries of Recruitment	The Netherlands
Health Condition(s) or Problem(s) Studied	Late preterm prelabour rupture of membranes (PROM between 34^{+0} and 36^{+6} weeks gestational age). Long-term effects of induction of labour versus expected management.
Intervention(s)	n/a
Key Inclusion and Exclusion Criteria	The PPROMEXIL Follow-up trial will analyse children of mothers with a singleton pregnancy (induction of labour n=359; expectant management n=353). At 10-12 years of (corrected) age all surviving children will be invited for follow-up.
Study Type	Follow-up of a randomized controlled trial
Date of First Enrollment	August 3rd, 2018

For peer review only - http://bmjopen.bmj.com/site/about/guidelines.xhtml

Sample Size	All sample size calculations are with a power of 90%, a two-sided a of 0.05 and ß of 0.20. To be able to detect a clinically relevant difference in mean scores of 0.5 SD in all tests, 86 children per group will be sufficient (total 172 children). This 0.5 SD equals a difference of 7.5 IQ points in the mean score of the WISC-V test (cognitive development), a difference of 1.5 points on the mean total standard scores of the M-ABC-2 (motor skills) and a difference of 5 points on the mean T scores in any of the broadband problem scales of the CBCL (behavioral development) between both groups. Thus, since 172 children comprise 24% of our total, also in case of limited follow up, differences of 0.5 SD can be picked up.
Recruitment Status	Open for patient inclusion
Primary Outcome(s)	Cognitive development (Wechsler Intelligence Scale for Children, WISC-V)<br><br>Motor skills (Movement-ABC-2)<br><br>Behaviour (Child Behavior Checklist, CBCL).
Key Secondary Outcomes	Academic attainment and behavior (Teacher Report Form, TRF)<br><br>Sensory processing (Short Sensory Profile, SSP)<br><br>Respiratory problems (International Study of Asthma and Allergies in Childhood questionnaire, ISAAC questionnaire)<br><br>Pubertal status (Puberty Developmental Scale, PDS)<br><br>Height, weight, bloodpressure<br><br>General health and demographics (questionnaires)
Ethics Review	The Medical Ethics Committee of the Academic Medical Centre Amsterdam (METC) has approved the PPROMEXIL Follow-up trial (METC 2016_217, NL58494.018.16).
Completion date	n/a
Summary Results	n/a
IPD sharing statement	n/a

Trial results will be submitted to a peer-reviewed journal, regardless of the outcome and made open access available in accordance with the Netherlands Organisation for Health Research and Innovation (ZonMW) policy. Results will be incorporated in national guidelines and patient information leaflets. Co-authorship will be based on the international committee

For peer review only - http://bmjopen.bmj.com/site/about/guidelines.xhtml

For peer review only

of medical journal editor's guidelines. Contributors that not fulfil these criteria will be listed as collaborators. The order of authors will be based on scientific input.

Availability of data and materials

The datasets used and/or analysed during the current study will be available from the corresponding author on reasonable request.

FOODNOTES

Author's contributions

AdR, NS, JvtH, AvWL, CAM, MvW, GJvB, FV, DvdH, ASPvT, BWM, TR, EP are member of the PPROMEXIL Follow-up trial study group and were involved in conception and design of the study. AdR, NS, JvtH drafted the manuscript which follows the SPIRIT checklist for reporting randomised trials. AdR, NS, JvtH, AvWL, CAM, MvW, GJvB, FV, DvdH, ASPvT, TR, BWM, EP discussed and fine-tuned the final design of the study. All authors edited the manuscript and read and approved the final version of the manuscript.

Funding statement

The study group received funding by ZonMW, the Netherlands Organization for Health Research and Development (governmental funding), grant number: 843002826. ZonMW peer reviewed the primary study protocol, they had no other involvement in study design. ZonMw will not have any involvement in data collection, nor in analysis or writing of the manuscript.

Competing interest statement

Dr. Ben Willem Mol is supported by a NHMRC Practitioner Fellowship (GNT1082548). Dr.

For peer review only - http://bmjopen.bmj.com/site/about/guidelines.xhtml

For peer review only

Ben Willem Mol reports consultancy for ObsEva, Merck Merck KGaA and Guerbet. All other authors did not report any conflicts of interest.

Acknowledgements

We would like to thank the research nurses and research midwives from the Dutch Consortium for Healthcare Evaluation and Research in Obstetrics and Gynecology - NVOG Consortium 2.0 as well as the Dutch Consortium Trialbureau for their efforts. The authors thank the contributors of PPROMEXIL and PPROMEXIL-2 trial. For a list of contributors to the PPROMEXIL and PPROMEXIL-2 trials see Additional file 3.

List of abbreviations

CBCL; Child Behavior Checklist, CI; confidence interval, CWIT; Color-Word Interference Test, DCD; Developmental Coordination Disorder, FSIQ; Full Scale IQ score, GA; gestational age, GBS; group B streptococci, IPDMA; Individual Participant Data Meta-analysis, IQ; intelligence quotient, ISAAC questionnaire; International Study of Asthma and Allergies in Childhood questionnaire, ISRCTN; International Standard Randomised Controlled Trial Number, M-ABC-2; Movement Assessment Battery for Children-2, M.D.; Doctor of Medicine, MEC; Medical ethics committee; in Dutch: medisch ethische toetsingscommissie (METC), NTR; Trial registration number, NVOG; the Dutch college of Obstetricians and Gynecologists, in Dutch: Nederlandse Vereniging voor Gynaecologie en Obstetrie (NVOG), PDS; Puberty Developmental Scale, PROM; prelabour rupture of membranes, PPROMEXIL trial; Preterm Prelabour Rupture Of Membranes EXpectant management vs Induction of Labour trial, PPROMT; the Preterm Pre-labour Rupture of Membranes close to Term Trial, RCT; randomised controlled trial, RR; relative risk, SD; standard deviation, SSP-NL; Short Sensory Profile, TRF; Teacher Report Form, WISC-V-NL; Wechsler Intelligence Scale for Children-V.

For peer review only

Figures and Tables

Figure 1. Overview of PPROMEXIL follow-up participants

Table 1. Chronology submission and revisions PPROMEXIL follow-up study

Table 2. WHO trial registration data set

Additional file 1. Overview of PPROMEXIL follow-up participants

Additional file 2. Schematic diagram of enrolment PPROMEXIL follow-up participants.

Additional file 3. Authors PPROMEXIL randomised controlled trials.

Additional file 4. Direct Acyclic Graph (DAG) figure

Additional file 5. GRIPP2 short checklist PPROMEXIL follow-up

For peer review only

REFERENCES

1. Nederlandse Vereniging voor Obstetrie en Gynaecologie [Rupture of membranes before onset of labour.] Available: http://nvog-documenten.nl/ index.php?pagina=/richtlijn/item/pagina.php&richtlijn_id= 564. Published: 2002. Accessed 19 November 2019.

2. van der Ham DP, van der Heyden JL, Opmeer BC, Mulder AL, Moonen RM, van Beek JH, et al. Management of late-preterm premature rupture of membranes: the PPROMEXIL-2 trial. Am J Obstet Gynecol. 2012;207(4):276 e1-10.

3. van der Ham DP, Vijgen SM, Nijhuis JG, van Beek JJ, Opmeer BC, Mulder AL, et al. Induction of labour versus expectant management in women with preterm prelabour rupture of membranes between 34 and 37 weeks: a randomisedrandomised controlled trial. PLoS Med. 2012;9(4):e1001208.

4. Morris JM, Roberts CL, Bowen JR, Patterson JA, Bond DM, Algert CS, et al. Immediate delivery compared with expectant management after preterm pre-labour rupture of the membranes close to term (PPROMT trial): a randomised controlled trial. Lancet. 2016;387(10017):444-52.

5. Quist-Nelson J, de Ruigh AA, Seidler AL, van der Ham DP, Willekes C, Berghella V, et al. Immediate Delivery Compared With Expectant Management in Late Preterm Prelabour Rupture of Membranes: An Individual Participant Data Meta-analysis. Obstet Gynecol. 2018;131(2):269-79.

6. Vijgen SM, van der Ham DP, Bijlenga D, van Beek JJ, Bloemenkamp KW, Kwee A, et al. Economic analysis comparing induction of labour and expectant management in women with preterm prelabour rupture of membranes between 34 and 37 weeks (PPROMEXIL trial). Acta Obstet Gynecol Scand. 2014;93(4):374-81.

7. Prelabour Rupture of Membranes: ACOG Practice Bulletin, Number 217. Obstet Gynecol. 2020;135(3):e80-e97.

8. Royal College of Obstetricians and Gynaecologists. Preterm prelabour rupture of membranes. Green-top guideline No. 44. London (UK): Royal College of Obstetricians and Gynaecologists; 2010.

9. van der Heyden JL, Willekes C, van Baar AL, van Wassenaer-Leemhuis AG, Pajkrt E, Oudijk MA, et al. Behavioural and neurodevelopmental outcome of 2-year-old children after preterm premature rupture of membranes: follow-up of a randomised clinical trial comparing induction of labour and expectant management. Eur J Obstet Gynecol Reprod Biol. 2015;194:17-23.

10. Armstrong-Wells J, Donnelly M, Post MD, Manco-Johnson MJ, Winn VD, Sebire G. Inflammatory predictors of neurologic disability after preterm premature rupture of membranes. Am J Obstet Gynecol. 2015;212(2):212 e1-9.

11. Chan AW, Tetzlaff JM, Gotzsche PC, Altman DG, Mann H, Berlin JA, et al. SPIRIT 2013 explanation and elabouration: guidance for protocols of clinical trials. BMJ. 2013;346:e7586.

For peer review only - http://bmjopen.bmj.com/site/about/guidelines.xhtml

12. https://www.castoredc.com/for-researchers/ Assessed on 19 November 2019

13. Wechsler D. Wechsler Intelligence Scale for Children, vijfde editie; Nederlandstalige bewerking. Amsterdam: Pearson Benelux B.V. 2018.

14. Henderson SE, Sugden DA. Movement Assessment Battery for Children: Manual. 1992: London: Psychological Corporation. 1992.

15. Verhulst VdE, Koot. Handleiding voor de CBCL/4-18. Rotterdam: Sophia Kinderziekenhuis, Erasmus MC. 1996.

16. Verhulst FC, Van der Ende J Handleiding voor de Teacher's Report Form (TRF). Rotterdam: Afdeling Kinder- en Jeugdpsychiatrie, Sophia Kinderziekenhuis / Erasmus MC. 2000.

17. Rietman A., Sensory Profile-NL. Handleiding. Pearson Assessment and Information, Amsterdam. 2013.

18. Asher MI, Keil U, Anderson HR, Beasley R, Crane J, Martinez F, et al. International Study of Asthma and Allergies in Childhood (ISAAC): rationale and methods. Eur Respir J. 1995;8(3):483-91.

19. Sole D, Camelo-Nunes IC, Wandalsen GF, Mallozi MC. Asthma in children and adolescents in Brazil: contribution of the International Study of Asthma and Allergies in Childhood (ISAAC). Rev Paul Pediatr. 2014;32(1):114-25.

20. Carskadon MA, Acebo C. A self-administered rating scale for pubertal development. J Adolesc Health. 1993;14(3):190-5.

21. Simons NE, van de Beek C, van der Lee JH, Opmeer BC, van Wassenaer-Leemhuis AG, van Baar AL, et al. Child outcomes after placement of a cervical pessary in women with a multiple pregnancy: A 4-year follow-up of the ProTWIN trial. Acta Obstet Gynecol Scand. 2019;98(10):1292-300.

22. van 't Hooft J, van der Lee JH, Opmeer BC, van Wassenaer-Leemhuis AG, van Baar AL, Bekedam DJ, et al. Pessary for prevention of preterm birth in twin pregnancy with short cervix: 3-year follow-up study. Ultrasound Obstet Gynecol. 2018;51(5):621-8.

23. van Os MA, van der Ven AJ, Kleinrouweler CE, Schuit E, Kazemier BM, Verhoeven CJ, et al. Preventing Preterm Birth with Progesterone in Women with a Short Cervical Length from a Low-Risk Population: A Multicenter Double-Blind Placebo-Controlled RandomisedRandomised Trial. Am J Perinatol. 2015;32(10):993-1000.

24. Hughes RA, Heron J, Sterne JAC, Tilling K. Accounting for missing data in statistical analyses: multiple imputation is not always the answer. Int J Epidemiol. 2019;48(4):1294-304.

25. Teune MJ, van Wassenaer AG, Malin GL, Asztalos E, Alfirevic Z, Mol BW, et al. Long-term child follow-up after large obstetric randomised controlled trials for the evaluation of perinatal interventions: a systematic review of the literature. BJOG. 2013;120(1):15-22.

For peer review only

26. van 't Hooft J, Duffy JM, Daly M, Williamson PR, Meher S, Thom E, et al. A Core Outcome Set for Evaluation of Interventions to Prevent Preterm Birth. Obstet Gynecol. 2016;127(1):49-58.

27. Kenyon S, Pike K, Jones DR, Brocklehurst P, Marlow N, Salt A, et al. Childhood outcomes after prescription of antibiotics to pregnant women with spontaneous preterm labour: 7-year follow-up of the ORACLE II trial. Lancet. 2008;372(9646):1319-27.

For peer review only

For peer review only - http://bmjopen.bmj.com/site/about/guidelines.xhtml

Figure 1. Overview of PPROMEXIL follow-up participants

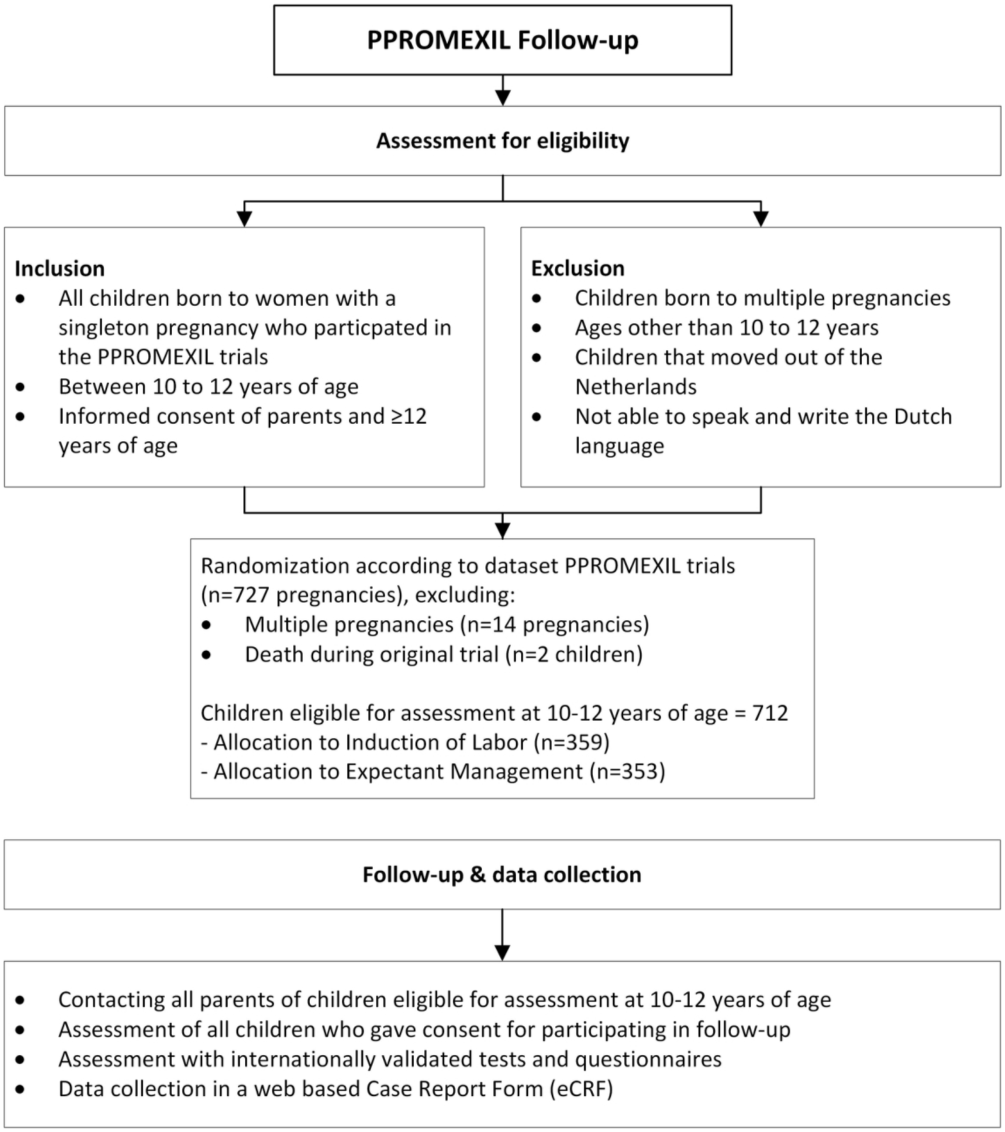

Overview of PPROMEXIL follow-up participants

144x170mm (300 x 300 DPI)

Additional file 1. SPIRIT checklist for reporting randomized trials

SPIRIT
Standard Protocol Items: Recommendations for Interventional Trials

Section/item	Item No	Description	Adressd on page number
**Administrative information**			
Title	1	Descriptive title identifying the study design, population, interventions, and, if applicable, trial acronym	1
Trial registration	2a	Trial identifier and registry name. If not yet registered, name of intended registry	5, 10, 24-25
	2b	All items from the World Health Organization Trial Registration Data Set	5, 10, 24-25, Table1
Protocol version	3	Date and version identifier	NA
Funding	4	Sources and types of financial, material, and other support	5, 24-26,
Roles and responsibilities	5a	Names, affiliations, and roles of protocol contributors	1-3, 26
	5b	Name and contact information for the trial sponsor	3
	5c	Role of study sponsor and funders, if any, in study design; collection, management, analysis, and interpretation of data; writing of the report; and the decision to submit the report for publication, including whether they will have ultimate authority over any of these activities	NA / 26
	5d	Composition, roles, and responsibilities of the coordinating centre, steering committee, endpoint adjudication committee, data management team, and other individuals or groups overseeing the trial, if applicable (see Item 21a for data monitoring committee)	NA

For peer review only

Introduction

Background and rationale	6a	Description of research question and justification for undertaking the trial, including summary of relevant studies (published and unpublished) examining benefits and harms for each intervention	7-9
	6b	Explanation for choice of comparators	7-9
Objectives	7	Specific objectives or hypotheses	8, 9
Trial design	8	Description of trial design including type of trial (eg, parallel group, crossover, factorial, single group), allocation ratio, and framework (eg, superiority, equivalence, noninferiority, exploratory)	10, 11

Methods: Participants, interventions, and outcomes

Study setting	9	Description of study settings (eg, community clinic, academic hospital) and list of countries where data will be collected. Reference to where list of study sites can be obtained	10-12
Eligibility criteria	10	Inclusion and exclusion criteria for participants. If applicable, eligibility criteria for study centres and individuals who will perform the interventions (eg, surgeons, psychotherapists)	10-12
Interventions	11a	Interventions for each group with sufficient detail to allow replication, including how and when they will be administered	13-17
	11b	Criteria for discontinuing or modifying allocated interventions for a given trial participant (eg, drug dose change in response to harms, participant request, or improving/worsening disease)	11
	11c	Strategies to improve adherence to intervention protocols, and any procedures for monitoring adherence (eg, drug tablet return, laboratory tests)	NA
	11d	Relevant concomitant care and interventions that are permitted or prohibited during the trial	NA

For peer review only

Outcomes	12	Primary, secondary, and other outcomes, including the specific measurement variable (eg, systolic blood pressure), analysis metric (eg, change from baseline, final value, time to event), method of aggregation (eg, median, proportion), and time point for each outcome. Explanation of the clinical relevance of chosen efficacy and harm outcomes is strongly recommended	13-17
Participant timeline	13	Time schedule of enrolment, interventions (including any run-ins and washouts), assessments, and visits for participants. A schematic diagram is highly recommended	10, 17, Figure1, Additional file 2
Sample size	14	Estimated number of participants needed to achieve study objectives and how it was determined, including clinical and statistical assumptions supporting any sample size calculations	17, 18
Recruitment	15	Strategies for achieving adequate participant enrolment to reach target sample size	11, 12
**Methods: Assignment of interventions (for controlled trials)**			
Allocation:			
Sequence generation	16a	Method of generating the allocation sequence (eg, computer-generated random numbers), and list of any factors for stratification. To reduce predictability of a random sequence, details of any planned restriction (eg, blocking) should be provided in a separate document that is unavailable to those who enrol participants or assign interventions	10-12
Allocation concealment mechanism	16b	Mechanism of implementing the allocation sequence (eg, central telephone; sequentially numbered, opaque, sealed envelopes), describing any steps to conceal the sequence until interventions are assigned	10-12
Implementation	16c	Who will generate the allocation sequence, who will enrol participants, and who will assign participants to interventions	10-12

Blinding (masking)	17a	Who will be blinded after assignment to interventions (eg, trial participants, care providers, outcome assessors, data analysts), and how	11-12
	17b	If blinded, circumstances under which unblinding is permissible, and procedure for revealing a participant's allocated intervention during the trial	NA
**Methods: Data collection, management, and analysis**			
Data collection methods	18a	Plans for assessment and collection of outcome, baseline, and other trial data, including any related processes to promote data quality (eg, duplicate measurements, training of assessors) and a description of study instruments (eg, questionnaires, laboratory tests) along with their reliability and validity, if known. Reference to where data collection forms can be found, if not in the protocol	12-17, 23
	18b	Plans to promote participant retention and complete follow-up, including list of any outcome data to be collected for participants who discontinue or deviate from intervention protocols	NA
Data management	19	Plans for data entry, coding, security, and storage, including any related processes to promote data quality (eg, double data entry; range checks for data values). Reference to where details of data management procedures can be found, if not in the protocol	12
Statistical methods	20a	Statistical methods for analysing primary and secondary outcomes. Reference to where other details of the statistical analysis plan can be found, if not in the protocol	18-20
	20b	Methods for any additional analyses (eg, subgroup and adjusted analyses)	19, 20
	20c	Definition of analysis population relating to protocol non-adherence (eg, as randomised analysis), and any statistical methods to handle missing data (eg, multiple imputation)	19, 20

Methods: Monitoring

Data monitoring	21a	Composition of data monitoring committee (DMC); summary of its role and reporting structure; statement of whether it is independent from the sponsor and competing interests; and reference to where further details about its charter can be found, if not in the protocol. Alternatively, an explanation of why a DMC is not needed	12
	21b	Description of any interim analyses and stopping guidelines, including who will have access to these interim results and make the final decision to terminate the trial	NA
Harms	22	Plans for collecting, assessing, reporting, and managing solicited and spontaneously reported adverse events and other unintended effects of trial interventions or trial conduct	NA
Auditing	23	Frequency and procedures for auditing trial conduct, if any, and whether the process will be independent from investigators and the sponsor	NA

Ethics and dissemination

Research ethics approval	24	Plans for seeking research ethics committee/institutional review board (REC/IRB) approval	10, 22-23
Protocol amendments	25	Plans for communicating important protocol modifications (eg, changes to eligibility criteria, outcomes, analyses) to relevant parties (eg, investigators, REC/IRBs, trial participants, trial registries, journals, regulators)	NA
Consent or assent	26a	Who will obtain informed consent or assent from potential trial participants or authorised surrogates, and how (see Item 32)	11, 23
	26b	Additional consent provisions for collection and use of participant data and biological specimens in ancillary studies, if applicable	NA

For peer review only

Confidentiality	27	How personal information about potential and enrolled participants will be collected, shared, and maintained in order to protect confidentiality before, during, and after the trial	12
Declaration of interests	28	Financial and other competing interests for principal investigators for the overall trial and each study site	24-27
Access to data	29	Statement of who will have access to the final trial dataset, and disclosure of contractual agreements that limit such access for investigators	26
Ancillary and post-trial care	30	Provisions, if any, for ancillary and post-trial care, and for compensation to those who suffer harm from trial participation	NA
Dissemination policy	31a	Plans for investigators and sponsor to communicate trial results to participants, healthcare professionals, the public, and other relevant groups (eg, via publication, reporting in results databases, or other data sharing arrangements), including any publication restrictions	11, 25-26
	31b	Authorship eligibility guidelines and any intended use of professional writers	26
	31c	Plans, if any, for granting public access to the full protocol, participant-level dataset, and statistical code	26
**Appendices**			
Informed consent materials	32	Model consent form and other related documentation given to participants and authorised surrogates	10, 11, 23
Biological specimens	33	Plans for collection, laboratory evaluation, and storage of biological specimens for genetic or molecular analysis in the current trial and for future use in ancillary studies, if applicable	NA

*It is strongly recommended that this checklist be read in conjunction with the SPIRIT 2013 Explanation & Elaboration for important clarification on the items. Amendments to the protocol should be tracked and dated. The SPIRIT checklist is copyrighted by the SPIRIT Group under the Creative Commons "Attribution-NonCommercial-NoDerivs 3.0 Unported" license

For peer review only

Additional file 2. Schematic diagram of schedule of enrolment, interventions, and assessments of the women participating in PPROMEXIL trials and children participating in PPROMEXIL follow-up

	STUDY PERIOD<br>Original PPROMEXIL trials - women			STUDY PERIOD<br>PPROMEXIL Follow-up - children		
	**Enrolment original trials**	**Allocation original trials**	**Outcomes original trials**	**Enrolment follow-up study**	**Assessment follow-up study**	**Close-out**
**TIMEPOINT**	*Women with PPROM between 34 and 36+6 weeks of gestation*	t = 0	*Pregnancy, childbirth and neonatal period*	*After 10 – 12 years*	*Age 10-12 years*	*Age 10-12 years*
**ENROLMENT:**						
**Eligibility screen**	X			X		
**Informed consent**	X			X	X	
**Allocation**		X				
**INTERVENTIONS:**						
**Induction of Labor**		X				
**Expectant Management**		X				
**ASSESSMENTS:**						
Baseline variables	X	X		X		
Outcome variables			X		X	X

Additional file 3.

Contributors to the PPROMEXIL and PPROMEXIL-2 trials

David P. van der Ham, Christine Willekes, Jantien L. van der Heyden, Sylvia M. C. Vijgen, Jan G. Nijhuis, Johannes J. van Beek, Brent C. Opmeer, Antonius L. M. Mulder, Rob Moonen, Mariët Groenewout, Mariëlle G. van Pampus, Gerald D. Mantel, Kitty W. M. Bloemenkamp, Wim J. van Wijngaarden, Marko Sikkema, Monique C. Haak, Paula J. M. Pernet, Martina Porath, Jan F. M. Molkenboer, Simone Kuppens, Anneke Kwee, Michael E. Kars, Mallory Woiski, Martin J. N. Weinans, Hajo I. J. Wildschut, Bettina M. C. Akerboom, Maureen T.M. Franssen, Christianne J.M. de Groot, J. Hans J. Duvekot, Bettina M.C. Akerboom, Aren J. van Loon, Jan W. de Leeuw, Bem Willem Mol, Aleid G. Leemhuis, E. Pajkrt, Martijn A. Oudijk, Bas Nij Bijvank, Caroline J. Bax, Janneke van ‘t Hooft.

For peer review only

Additional file 4. Direct Acyclic Graph (DAG) identifying potential confounding measures

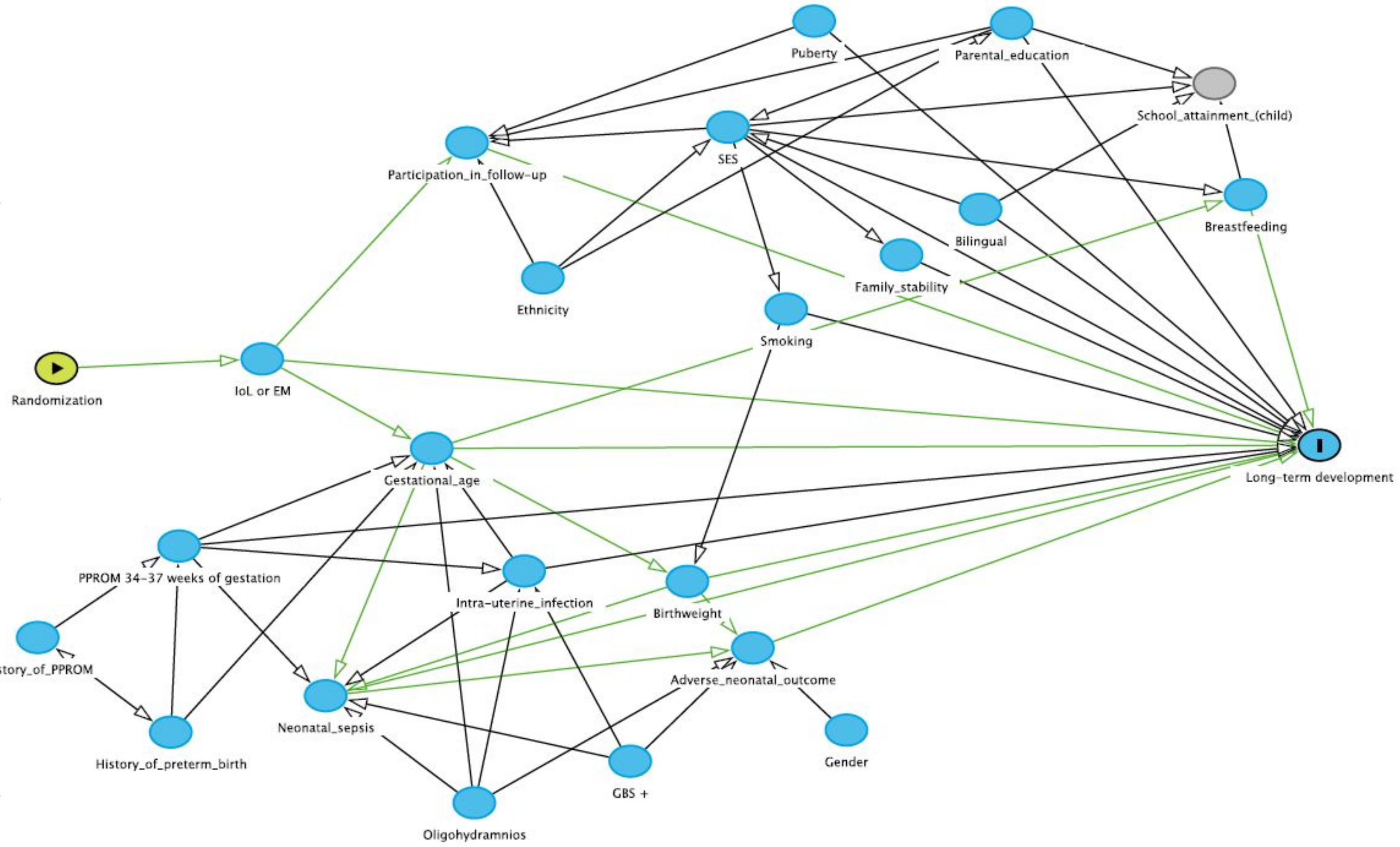

For peer review only

Additional file 5. GRIPP2 short form

Section and topic	Item	Reported on page No
1: Aim	Report the aim of PPI in the study	8, 20-21
2: Methods	Provide a clear description of the methods used for PPI in the study	8, 20-21
3: Study results	Outcomes—Report the results of PPI in the study, including both positive and negative outcomes	8
4: Discussion and conclusions	Outcomes—Comment on the extent to which PPI influenced the study overall. Describe positive and negative effects	NA
5: Reflections/critical perspective	Comment critically on the study, reflecting on the things that went well and those that did not, so others can learn from this experience	NA

For peer review only

BMJ Open

Child outcomes after induction of labour or expectant management in women with preterm prelabour rupture of membranes between 34-37 weeks of gestation: Study protocol of the PPROMEXIL Follow-up trial. A long-term follow-up study of the randomised controlled trials PPROMEXIL and PPROMEXIL-2.

Journal:	*BMJ Open*
Manuscript ID	bmjopen-2020-046046.R1
Article Type:	Protocol
Date Submitted by the Author:	28-Mar-2021
Complete List of Authors:	de Ruigh, Annemijn; Amsterdam UMC Location AMC, Department of Obstetrics and Gynaecology, Amsterdam Reproduction & Development<br>Simons, Noor; Amsterdam UMC Location AMC, Department of Obstetrics and Gynaecology, Amsterdam Reproduction & Development<br>Van 't Hooft, Janneke; Amsterdam UMC Location AMC, Department of Obstetrics and Gynaecology, Amsterdam Reproduction & Development<br>van Wassenaer-Leemhuis, Aleid; Amsterdam UMC Location AMC, Department of Neonatology and Paediatrics, Emma Children's Hospital, Amsterdam Reproduction & Development<br>Aarnoudse-Moens, Cornelieke; Amsterdam UMC Location AMC, Department of Neonatology and Paediatrics, Emma Children's Hospital, Amsterdam Reproduction & Development<br>van Wely, Madelon; Amsterdam University Medical Centres, Netherlands Satellite of the Cochrane Gynaecology and Fertility Group<br>van Baaren, Gert-Jan; Amsterdam UMC Location AMC, Department of Obstetrics and Gynaecology<br>Vlemmix, Floortje; Amsterdam UMC Location AMC, Department of Obstetrics and Gynaecology<br>van der Ham, D.P.; Martini Hospital, Department of Obstetrics and Gynaecology<br>van Teeffelen, Augustinus; Maastricht UMC+, Department of Obstetrics and Gynaecology<br>Mol, Ben; Monash University, Department of Obstetrics and Gynaecology<br>Roseboom, Tessa; Amsterdam UMC Location AMC, Department of Obstetrics and Gynaecology, Amsterdam Reproduction & Development; Amsterdam UMC Locatie AMC, Department of Clinical Epidemiology, Biostatistics and Bioinformatics,<br>Pajkrt, Eva; Amsterdam UMC Location AMC, Department of Obstetrics and Gynaecology, Amsterdam Reproduction & Development
**Primary Subject Heading**:	Obstetrics and gynaecology
Secondary Subject Heading:	Paediatrics
Keywords:	Fetal medicine < OBSTETRICS, Maternal medicine < OBSTETRICS,

	Prenatal diagnosis < OBSTETRICS, Developmental neurology & neurodisability < PAEDIATRICS

SCHOLARONE™
Manuscripts

I, the Submitting Author has the right to grant and does grant on behalf of all authors of the Work (as defined in the below author licence), an exclusive licence and/or a non-exclusive licence for contributions from authors who are: i) UK Crown employees; ii) where BMJ has agreed a CC-BY licence shall apply, and/or iii) in accordance with the terms applicable for US Federal Government officers or employees acting as part of their official duties; on a worldwide, perpetual, irrevocable, royalty-free basis to BMJ Publishing Group Ltd ("BMJ") its licensees and where the relevant Journal is co-owned by BMJ to the co-owners of the Journal, to publish the Work in this journal and any other BMJ products and to exploit all rights, as set out in our licence.

The Submitting Author accepts and understands that any supply made under these terms is made by BMJ to the Submitting Author unless you are acting as an employee on behalf of your employer or a postgraduate student of an affiliated institution which is paying any applicable article publishing charge ("APC") for Open Access articles. Where the Submitting Author wishes to make the Work available on an Open Access basis (and intends to pay the relevant APC), the terms of reuse of such Open Access shall be governed by a Creative Commons licence – details of these licences and which Creative Commons licence will apply to this Work are set out in our licence referred to above.

Other than as permitted in any relevant BMJ Author's Self Archiving Policies, I confirm this Work has not been accepted for publication elsewhere, is not being considered for publication elsewhere and does not duplicate material already published. I confirm all authors consent to publication of this Work and authorise the granting of this licence.

For peer review only

Child outcomes after induction of labour or expectant management in women with preterm prelabour rupture of membranes between 34-37 weeks of gestation: Study protocol of the PPROMEXIL Follow-up trial

A long-term follow-up study of the randomised controlled trials PPROMEXIL and PPROMEXIL-2.

Annemijn A. de Ruigh, MD
Department of Obstetrics and Gynaecology, Academic Medical Centre (AMC), Amsterdam, The Netherlands

Noor E. Simons, MD
Department of Obstetrics and Gynaecology, Academic Medical Centre (AMC), Amsterdam, The Netherlands

Janneke van ‘t Hooft, MD PhD
Department of Obstetrics and Gynaecology, Academic Medical Centre (AMC), Amsterdam, The Netherlands

Aleid G. van Wassenaer-Leemhuis, MD PhD
Department of Neonatology and Paediatrics, Emma Children's Hospital, Academic Medical Center, Amsterdam, The Netherlands.

Cornelieke S.H. Aarnoudse-Moens, PhD
Department of Neonatology and Paediatrics, Emma Children's Hospital, Academic Medical

For peer review only - http://bmjopen.bmj.com/site/about/guidelines.xhtml

Center, Amsterdam, The Netherlands.

Madelon van Wely, PhD

Centre for Reproductive Medicine, Academic Medical Center, Amsterdam, the Netherlands

Gert-Jan van Baaren MD PhD

Department of Obstetrics and Gynaecology, Academic Medical Center, Amsterdam, The Netherlands.

Floortje Vlemmix MD PhD

Department of Obstetrics and Gynaecology, Academic Medical Center, Amsterdam, The Netherlands.

David P van der Ham, MD, PhD

Department of Obstetrics and Gynaecology, Martini Hospital, Groningen, The Netherlands.

A.S.P. van Teeffelen, MD PhD

Department of Obstetrics and Gynaecology, GROW - School for Oncology and Developmental Biology, Maastricht University Medical Centre, Maastricht, The Netherlands.

Ben Willem Mol, MD PhD

Obstetrics and Gynaecology, Monash University, Melbourne, Victoria, Australia

Tessa J. Roseboom, PhD

Department of Clinical Epidemiology, Biostatistics and Bioinformatics, Academic Medical Centre, University of Amsterdam, Amsterdam, The Netherlands; Department of Obstetrics and Gynaecology, Academic Medical Centre, University of Amsterdam, The Netherlands.

For peer review only

Eva Pajkrt, MD, PhD

Department of Obstetrics and Gynaecology, Academic Medical Centre (AMC), Amsterdam, The Netherlands

Word count: Total word count main text: 4,403

Disclosure: Dr. Ben Willem Mol is supported by a NHMRC Practitioner Fellowship (GNT1082548). Dr. Ben Willem Mol reports consultancy for ObsEva, Merck Merck KGaA and Guerbet. All other authors did not report any conflicts of interest.

Financial Support: This study is funded by ZonMW (grant number 843002826).

Corresponding Author: Annemijn A. de Ruigh, MD, a.a.deruigh@amsterdamumc.nl

Department of Obstetrics and Gynaecology, Academic Medical Center (AMC), Meibergdreef 9, 1105, AZ, Amsterdam, The Netherlands

For peer review only

ABSTRACT

Introduction: Late preterm prelabour rupture of membranes (PROM between 34^{+0} and 36^{+6} weeks gestational age) is an important clinical dilemma. Previously, two large Dutch randomised controlled trials (RCTs) compared induction of labour (IoL) to expectant management (EM). Both trials showed that early delivery does not reduce the risk of neonatal sepsis as compared to EM, although prematurity related risks might increase. An extensive, structured long-term follow-up of these children has never been performed.

Methods and analysis: The PPROMEXIL Follow-up trial aims to assess long-term childhood outcomes of the PPROMEXIL (ISRCTN29313500) and PPROMEXIL-2 trial (ISRCTN05689407), two multicentre RCTs using the same protocol, conducted between 2007-2010 evaluating IoL versus EM in women with late preterm PROM. The PPROMEXIL Follow-up will analyse children of mothers with a singleton pregnancy (PPROMEXIL trial n= 520, PPROMEXIL-2 trial n=191, total IoL n=359; total EM n=352). At 10-12 years of age all surviving children will be invited for a neurodevelopmental assessment using the Wechsler Intelligence Scale for Children-V, Color-Word Interference Test and the Movement Assessment Battery for Children-2. Parents will be asked to fill out questionnaires assessing behaviour, motor function, sensory processing, respiratory problems, general health and need for health care services. Teachers will fill out the Teacher Report Form and answer questions regarding school attainment. For all tests means with SD's will be compared, as well as predefined cut-off scores for abnormal outcome. Sensitivity analyses consisting of different imputation techniques will be used to deal with loss-to-follow-up.

Ethics and dissemination: The study has been granted approval by the MEC of the AmsterdamUMC (MEC2016_217). Results will be disseminated through peer-reviewed journals and summaries shared with stakeholders. This protocol is published before analysis of the results.

For peer review only - http://bmjopen.bmj.com/site/about/guidelines.xhtml

Registration: Dutch Trial registration number: NTR6953 (registration December 28th, 2017).

The study has been peer reviewed, approved and funded by ZonMW (843002826).

Key words: Late preterm prelabour rupture of membranes, induction of labour, expectant management, long-term outcome, child development, child health

For peer review only - http://bmjopen.bmj.com/site/about/guidelines.xhtml

ARTICLE SUMMARY

Strengths and limitations of this study

- This long-term follow-up study will be the first study to evaluate long-term developmental outcomes (cognitive, motor, and behavioural development, sensory processing, respiratory problems, general health, children's need for health-care services, and school attainment) in the offspring of women who have been treated during pregnancy with induction of labour or expectant management for late preterm prelabour rupture of membranes.
- Children will be evaluated at 10-12 years of age with internationally validated neurodevelopmental tests by a trained team consisting of a (neuro)psychologist and physician masked to the study group, and with questionnaires, translated for Dutch children, using norm scores for Dutch children.
- The study will be performed within the Dutch Consortium for Healthcare Evaluation and Research in Obstetrics and Gynecology - NVOG Consortium 2.0, a collaboration of approximately 70 obstetric hospitals (academic and non-academic hospitals) in the Netherlands.
- Alongside this long-term follow-up study a separately reported economic evaluation study will be planned to investigate cost-effectiveness of both treatments taking long-term developmental outcomes into account.
- The main limitation is that we expect to have an incomplete follow-up rate due to a high loss to follow-up, which we estimate to be 60 to 70%. Baseline characteristics of children participating in follow-up versus lost to follow-up will be compared, to assess whether selection or attrition bias may be present in our study.

For peer review only

INTRODUCTION

Background and rationale

Late preterm prelabour rupture of membranes (late preterm PROM) between 34^{+0} and 36^{+6} weeks gestation, is an important clinical problem occurring in 1.5% of pregnant women, of which 25% will deliver within 24 hours.(1) After PROM, the risk of infection increases for both mother and foetus. Recently, three large randomised controlled trials (RCTs) compared induction of labour to expectant management for women whose pregnancy was complicated by late preterm PROM.(2-4) The Dutch PPROMEXIL and PPROMEXIL-2 trial, and the Australian PPROMT trial showed that induction of labour does not reduce the risk of neonatal sepsis as compared to expectant management, while increasing prematurity related risks, such as hypoglycaemia and hyperbilirubinemia. Furthermore, an Individual Participant Data Meta-analysis (IPD-MA) investigating participant data of all three RCTs also concluded that expectant management is an acceptable alternative to induction of labour, as both treatments resulted in comparable rates of a composite of adverse neonatal outcomes.(5) Moreover, an economic analysis of the PPROMEXIL trial, showed that health care costs for induction of labour are slightly higher, although not statistically significant, with a mean difference of €754 (€8,094 for induction of labour versus €7,340 for expectant management, 95% confidence interval (CI) -€335 to €1,802).(6) Therefore, currently most national guidelines advocate expectant management for late preterm PROM.(1, 7, 8)

In 2015 our research team performed a follow-up study of children at two years of age, born to women who participated in the PPROMEXIL trial.(9) This follow-up study was performed with limited budget and used internationally validated screening questionnaires. Even though this study had a follow-up rate of 44% and no extensive neurodevelopmental assessments were used, an increase in neurodevelopmental impairment was found in the expectant management group as compared to the induction of labour group (abnormal score (-2 standard deviation (SD)) in ≥1 domains of the Ages and Stages Questionnaire: 14%

For peer review only - http://bmjopen.bmj.com/site/about/guidelines.xhtml

induction of labour group versus 26% expectant management group, difference in percentage -11.4; 95% CI -21.9 to -0.98).(9) Hypothetically, a prolonged stay of the foetus in an environment at risk for (subclinical) infections such as maternal placental inflammation (histological or clinical chorioamnionitis) and foetal side placental inflammation (funisitis and chorionic plate vasculitis) in case of expectant management could affect brain development (i.e. neurological outcome) and therefore explain the neurodevelopmental impairment seen at 2 years of age.(10) The developmental effects of induction of labour or expectant management after late preterm PROM in children after 2 years are still unknown. Furthermore, understanding the long-term effects on women's offspring of either treatment is important for both clinicians and pregnant women when deciding how to manage late preterm PROM.

Until now, no other study has performed or planned a comprehensive long-term follow-up of children born after late preterm PROM. Study feasibility was investigated by an online questionnaire filled out by parents and members of a Dutch patient organization representing patients affected by preterm birth due to complications in pregnancy. Results showed that 89% of parents were willing to participate in an extensive follow-up study. Parents rated the outcomes general health, behaviour, school attainment and respiratory problems as most important outcomes (data not published). A systematic review on neurodevelopment in preterm children showed a strong relationship between gestational age at delivery and cognitive abilities (i.e. academic attainment, emotional and behavioural needs) in very, moderately and late preterm infants. These deficits persist beyond primary school for all neurodevelopmental domains. They stress the importance of knowledge on these long-term domains and advise trials to plan long-term follow-up to gain insight on possible neurodevelopmental delay in children.(11)

Objectives

Therefore, the aim of this study is to conduct a structured follow-up of all children born to

For peer review only - http://bmjopen.bmj.com/site/about/guidelines.xhtml

women with late preterm PROM who were randomised to induction of labour or expectant management in the PPROMEXIL and PPROMEXL-2 trial. Long-term cognitive, motor, and behavioural development, sensory processing, respiratory problems, general health, children’s need for health-care services, and school attainment will be assessed at 10-12 years of age using internationally validated measurements and questionnaires, translated and using norm scores for Dutch children.

For peer review only

For peer review only - http://bmjopen.bmj.com/site/about/guidelines.xhtml

METHODS AND ANALYSIS

Study setting

We will perform an extensive long-term follow-up study of two previously executed RCTs (PPROMEXIL Follow-up trial, NTR 6953, METC 2016_217, NL58494.018.16) investigating long-term developmental outcomes (cognitive, motor, behavioural development), sensory processing, respiratory problems, general health, children's need for health-care services, and school attainment. This will be assessed at 10-12 years of corrected age in children born to women with a singleton pregnancy complicated by late preterm PROM (between 34+0 and 36+6 weeks gestation), who participated in the RCTs PPROMEXIL, and PPROMEXIL-2 trial. Details of the PPROMEXIL (ISRCTN29313500) and PPROMEXIL-2 trial (amendment of the PPROMEXIL trial (MEC 05-240, ISRCTN05689407) have been published elsewhere.(2, 3) These two large RCTs, using the same study protocol and conducted between 2007 and 2011 in 61 academic and non-academic hospitals in The Netherlands, assessed whether induction of labour versus expectant management would reduce the incidence of neonatal sepsis in women with late preterm PROM. In the induction of labour group, patients were induced within 24 hours after randomization. Patients in the expectant management group were monitored until the onset of spontaneous delivery or induced after 37+0 weeks according to national guidelines.(1)

Participants and eligibility criteria

All children born to women with a singleton pregnancy who participated in the PPROMEXIL trials will be invited for this long-term follow-up assessment. Children will be evaluated at 10-12 years of age. As the total number of multiple pregnancies in the PPROMEXIL- and PPROMEXIL-2 trials was very low (14/727 (1.9%) and equally distributed among treatment groups), only singleton pregnancies will be included in the analysis. See Figure 1. for the overview of PPROMEXIL follow-up participants.

For peer review only - http://bmjopen.bmj.com/site/about/guidelines.xhtml

Procedures and recruitment

The study protocol is designed, constructed and reported according to the recommendations given in the Standard Protocol Items: Recommendations for Interventional Trials (See Additional file 1. SPIRIT checklist for reporting randomised trials, Additional file 2. SPIRIT schematic diagram of enrolment PPROMEXIL follow-up participants., and Additional file 3. GRIPP2 short form.)(12) The study will be performed within the Dutch Consortium for Healthcare Evaluation and Research in Obstetrics and Gynecology - NVOG Consortium 2.0, a collaboration of approximately 70 obstetric hospitals (academic and non-academic hospitals) in the Netherlands (https://zorgevaluatienederland.nl/nvog). Research nurses will be asked to crosscheck medical records for possible occurrence of death of women's offspring before contacting parents and their child for participating in this follow-up study. All parents will be contacted by post to announce this follow-up study, and if they give consent to be approached by the research team, they will be contacted by telephone or email to explain study details. Parents will be informed that participation is voluntary and that they may withdraw consent to participate at any time (see Additional file 4). They will be informed that declining participation will not affect their or their child's care. Parents will be given sufficient time to read the patient information and they will be given the opportunity to ask questions by telephone or email prior to signing the informed consent form. Written study information at children's reading level will be available for all children (specified for children <12 years of age and ≥12 years of age. An independent physician (i.e. not a member of the research team) will be available to answer any questions patients may have. Written informed consent will be obtained from both parents prior to the examination. Children ≥12 years of age have to sign their own informed consent, in addition to the informed consent of their parents, at the day of the assessment. A copy of the informed consent form(s) will be given to the parents/child. All study documents will be available through the study website.

Concealment of treatment allocation at time of the PPROMEXIL and PPROMEXIL-2 trials (i.e. induction of labour or expectant management) was not possible due to the type of

For peer review only - http://bmjopen.bmj.com/site/about/guidelines.xhtml

intervention, and therefore parents and children entered in this follow-up study will be aware of treatment allocation. The research team performing the follow-up examinations and all members of the research team performing data entry and data analyses will be masked to treatment allocation.

All data will be collected, captured, and coded in accordance with existing polices to ensure patient confidentiality. Data will be recorded using an electronic case record form and will be stored in a web-secured database (available through the study website).(13) The investigators will publish the results of this trial in a peer reviewed medical journal as soon as appropriate. The Clinical Research Unit (CRU) of the Amsterdam UMC will monitor data collection.

Follow-up assessment and outcomes

During a single visit in an outpatient clinic of a local hospital close to the family's neighbourhood, children will be assessed on long-term neurodevelopmental outcomes using standardized and validated neurodevelopmental tests and questionnaires. A trained team consisting of a (neuro)psychologist and physician, masked to the study group, will perform all neurodevelopmental tests. Neurodevelopmental assessment of children has a structured approach, is enjoyable for most children and is not invasive. During neurodevelopmental assessment of the child, parents will be asked to fill out questionnaires on sensory processing, behaviour, respiratory problems, and child's health. If necessary, parents will be assisted with filling out questionnaires. All, but one, questionnaires are digital and can be filled out on a tablet during the assessment or at any other time at home.

After completing all examinations, parents receive a short report on their child's test results. This short report will give information on total test scores and tell parents whether their child's scores are above, on or below average. If test scores indicate that children would benefit from supportive (health, developmental or educational) care, parents are advised to contact

For peer review only - http://bmjopen.bmj.com/site/about/guidelines.xhtml

their general practitioner for referral to a paediatrician or psychologist.

Main study outcomes

- Cognitive development (Wechsler Intelligence Scale for Children -V)
- Motor skills (Movement-ABC-2)
- Behaviour (Child Behaviour Checklist)

Secondary study outcomes

- School attainment (Teacher's Report Form and additional questions)
- Sensory processing (Short Sensory Profile-NL)
- Respiratory problems (International Study of Asthma and Allergies in Childhood questionnaire)
- Pubertal status (Puberty Developmental Scale)
- General health (questionnaire)

Assessment of cognitive development

Cognitive Development will be assessment using the Dutch version of the Wechsler Intelligence Scale for Children (WISC-V).(14) The WISC-V is used worldwide to assess cognition in children aged six to 16 years and consists of 10 subtests that are combined into a Full Scale IQ score (FSIQ) and five primary indexes: verbal comprehension, visual spatial, fluid reasoning, working memory and processing speed. Besides these primary indexes, an additional mathematics subtest will be obtained to provide an objective measurement of this area of academic attainment. The WISC-V total intelligence quotient (IQ) score and primary indexes have a mean score of 100 points with a SD of 15 points. We will compare mean (SD) between treatment groups. Furthermore, an index score ≤70 (≥ -2 SD below the mean score) will be considered as a severe cognitive delay and will be compared between groups. An index score >70 and ≤84 (≥ -1 SD and < -2SD below the mean score) will indicate a mild

For peer review only - http://bmjopen.bmj.com/site/about/guidelines.xhtml

cognitive delay. Normal cognitive outcome is defined as no severe or mild neurodevelopmental delay. A difference between the two treatment groups of 7.5 points (0.5 SD) could indicate a potential clinical relevant difference.

Child's executive functioning will be tested using subtests of the WISC-V and the Color-Word Interference Test (CWIT). The CWIT measures cognitive set shifting and the ability to inhibit a dominant and automatic verbal response by separate and combined Color Naming and Color Reading items. The CWIT subtests have a mean of 10 points with a SD of 2 points. An CWIT index score of ≤4 (i.e. more than -2 SD below the mean score) is considered a severe delay in executive functioning and will be analysed.

Assessment of motor skills

Child's motor function will be measured by the Movement Assessment Battery for Children-2 (M-ABC-2).(15) The M-ABC-2 is the most commonly used tool used to examine fine and gross motor skills. The M-ABC-2 provides data about a child's performance of age-appropriate tasks within three domains; manual dexterity, aiming and catching, and balance. M-ABC-2 scores will be calculated as standard scores and percentiles for each domain, and as a total test score. The mean standard score for all domains and the total score is 10 points, with a SD of 3 points. We will compare mean (SD) between treatment groups. The age band two (7-10 years of age) and three (11-16 years of age) of the M-ABC-2 will be used, as appropriate according to the child's age. Percentiles as defined by the M-ABC-2 testing manual and used in daily practice for testing motor skills in children will be applied. In short, a standard score of ≤5 points, representing ≤5th percentile will be defined as a significant movement difficulty and a severe delay in motor skills and will be compared between treatment groups. A standard score of 6 or 7 points, representing >5th to ≤16th percentile will indicate that the child is at risk of having a movement difficulty and therefore will be classified as mild delay in motor skills. A standard score of ≥8 points, representing>16th percentile will be defined as no movement difficulty and normal development of motor skills.

For peer review only - http://bmjopen.bmj.com/site/about/guidelines.xhtml

Additionally, parents will fill out the M-ABC-2 checklist, a questionnaire that consists of three sections on movement in static and/or predictable environment, movement in a dynamic and/or unpredictable environment and non-motor factors that may affect the child's movement. The sections on static and dynamic movements are summed up to a total score, with a higher score indicating a worse motor function. A total score of $\geq 95^{th}$ percentile (≥ 9 points) indicates severe motor impairment and will be compared between both treatment groups.

Assessment of behavioural development

Child's behaviour will be measured by the Child Behaviour Checklist (CBCL), a parental questionnaire used to screen for behaviour problems in children.(16) It informs on eight narrow syndrome scales (anxious/depressed, withdrawn/depressed, somatic complaints, social problems, thought problems, attention problems, rule-breaking behaviour, and aggressive behavior) and three broadband scales (internalizing, externalizing behavioural problems and a total problems score) which are composed out of the narrow-band syndrome scales. The CBCL broadband scales T scores have a mean of 50 points with a SD of 10 points. We will compare mean (SD) between treatment groups. Furthermore, a score $>90^{th}$ percentile (>63 points) on one of the two broad dimensions scales (internalizing problems or externalizing problems), or the total problem score (sum of all scores) of the CBCL will be defined as abnormal and clinically relevant for indicating behavioural problems. Scores $\geq 84^{st}$ and $\leq 90^{th}$ percentile (≥ 60 and ≤ 63 points) are considered borderline and scores $<84^{th}$ percentile (<60 points) are defined as normal.

Assessment of school attainment

Child's academic attainment and behaviour will be assessed using the Teacher's Report Form (TRF).(17) The TRF assesses problem behaviour in the last two months and identifies the same eight syndromes as the CBCL, and also inquires on academic attainment (Academic Performance). With parental permission, the TRF will be filled out by the child's school teacher (the teacher who has known the child in the school setting for more than two

For peer review only

For peer review only - http://bmjopen.bmj.com/site/about/guidelines.xhtml

months can complete the TRF). Accompanying the TRF, teachers will be asked some additional questions regarding the child's need for additional educational support in- or outside the classroom. For the TRF the cut-off percentiles of the broad band and total scores as used in the CBCL will be applied. For Academic Performance a cut-off score of <10th percentile (≤36 points) will be defined as abnormal. Scores between 10th and 16th percentile are classified as borderline and ≥17th percentile are considered normal outcome.

Assessment of sensory processing

Sensory processing will be determined using the Short Sensory Profile questionnaire (SSP-NL).(18) The Short Sensory Profile contains sections corresponding to each sensory system, sections that indicate the modulation of sensory input across sensory systems, and sections that indicate behavioural and emotional responses that are associated with sensory processing. This questionnaire consists of 38 items, classified into seven subscales (Tactile Sensitivity, Taste/Smell Sensitivity, Movement Sensitivity, Underresponsive/Seeks Sensation, Auditory Filtering, Low Energy/Weak, and Visual/Auditory Sensitivity). For every subscale parents will be asked how frequently their children respond in the way described by each item using a 5 point Likert scale (nearly never, seldom, occasionally, frequently, almost always). Lower scores on the total score and subscales indicate more sensory symptoms. Subscales and the total scores will be used to classify as “definite difference” (cut off scores ≥-2 SD below the mean) and will be compared between groups. “Typical performance” will be defined as < -1 SD below the mean, “probable difference” will be defined as ≥ -1 SD and < -2 SD below the mean.

Assessment of respiratory problems

Respiratory problems, such as asthma or other lung problems will be assessed using the International Study of Asthma and Allergies in Childhood questionnaire (ISAAC questionnaire) which informs on asthma, rhinitis and eczema.(19) The diagnosis of asthma will be defined as a positive answers to the question: “In the last 12 (twelve) months, has your child had wheezing?”, as this question has a sensitivity of 100%, specificity of 78%,

For peer review only - http://bmjopen.bmj.com/site/about/guidelines.xhtml

positive predictive value of 73%, and negative predictive value of 100% for the diagnosis of asthma.(20)

Assessment of anthropometry and pubertal status

Children will be asked to fill out the Puberty Developmental Scale (PDS), a self-report measure of pubertal status.(21) Children will be asked questions regarding on e.g. growth in height, skin changes, body or facial hair, deepening of the voice (for boys), and starting to menstruate or developing breasts (for girls). Physical examination will be restricted to measurement of height/weight and blood pressure. Results of physical examination (height/weight, body mass index) will be used for baseline characteristics. Puberty status will be used for baseline characteristics and subgroup analysis.

Assessment of child's health and need for health care services

A general questionnaire consisting of 61 items, will be used to assess demographic characteristics and will ask questions regarding the present (last 12 months) and the past health and health care use (from discharge after delivery until date of assessment) (also used in previous follow-up studies such as ProTWIN*kids* study at three and four years, TripleP study(22-24)). Questions address child's health, need for health care services, hospital visits, hospital submission, need for surgery, use of medication, psychological problems, need for developmental therapies (such as physical therapists, remedial teaching, speech therapist, occupational therapist). Health care use and (health) related problems will be clustered into different clinically relevant groups (e.g. need for medical specialist and/or developmental care, medication use in the past and present, hospital admissions and surgery to give insight in the range of health related problems).

Parents will be asked to give permission to gather medical information on the child's health via the general practitioner and the preventive youth healthcare services if needed.

Economic analysis

Alongside this long-term follow-up study, an economic evaluation study will be planned to

For peer review only

For peer review only - http://bmjopen.bmj.com/site/about/guidelines.xhtml

investigate cost-effectiveness of both treatments taking long-term developmental outcomes into account. Results of this economic evaluation will be reported separately from trial results.

At present, no additional long-term follow-up in later life (>12 years of age) is planned. Permission to approach parents and children for additional follow-up research in later life will be obtained with informed consent form during the current follow-up study. If additional long-term follow-up of children at an adolescence age will be planned in the future, additional approval of the Medical Research Ethics Committee will be sought.

Sample size

Since this is a follow up study, the maximum number of study participants is already defined by the two PPROMEXIL trials, excluding multiple pregnancies and deceased children (Figure 1 and Additional file 2.). Consequently, 711 children are eligible for inclusion, 359 born in the induction of labour group and 352 born in the expectant management group (PPROMEXIL trial n=520, PPROMEXIL-2 trial n=191). As we will not be able to adjust the number of recruited children, a power calculation will not be of any use to calculate a study sample size. However, this calculation can indicate the minimum number of children that need to be tested in order to find a clinically significant difference for the three main study outcomes: cognitive development, motor skills and behavioural development. All sample size calculations are with a power of 90%, a two-sided a of 0.05 and ß of 0.20. To be able to detect a clinically relevant difference in mean scores of 0.5 SD in the main outcomes, minimally 86 children per group are needed (total 172 children). This 0.5 SD equals a difference of 7.5 IQ points in the mean score of the WISC-V test (cognitive development), a difference of 1.5 points on the mean total standard scores of the M-ABC-2 (motor skills) and a difference of 5 points on the mean T scores in any of the broadband problem scales of the CBCL (behavioral development) between both groups. Thus, since 172 children comprise 24% of our total, also in case of

For peer review only - http://bmjopen.bmj.com/site/about/guidelines.xhtml

limited follow up, differences of 0.5 SD can be picked up. Based on previous experience in our research team with follow-up trials and based of existing literature, we expect to have a follow-up rate of 30 to 40% of the children.(25)

Statistical methods

Differences in background characteristics and the maternal, pregnancy, delivery and neonatal outcomes between the induction of labour group and expectant management group will be compared using unpaired T-test, Mann-Whitney U test, Chi-square test or Fisher's exact test when appropriate. The same characteristics will be compared in children assessed at follow-up and for the original participants of the PPROMEXIL trials. This will allow us to assess whether selection or attrition bias may be present in our study (e.g. due to drop-out of healthy or unhealthy children). To compare the long-term developmental outcomes between both treatment groups mean differences and the corresponding 95% CI will be calculated. For dichotomous outcomes relative risk (RR) with corresponding 95% CI will be calculated. Our main analyses shall be based upon the results from the children assessed in follow-up (complete case analysis).(25)

The relatively simple statistical analysis described above can be justified by the fact that our study is a follow-up of two RCTs and consequently no confounding measures are expectant (See Additional file 5. Direct Acyclic Graph (DAG)). The DAG confirms that there are no variables susceptive to have influenced the likelihood of receiving the intervention and subsequently have influenced long-term outcome of the child. On the other hand, selection bias may occur as a consequence of incomplete follow-up. We will evaluate the effect of differences in background characteristics (such as maternal smoking, social-economic status) and if applicable, report unadjusted and adjusted odds ratios for dichotomous outcomes using logistic models and adjusted mean differences and the corresponding 95% CI for continuous outcomes using general linear models.

For peer review only - http://bmjopen.bmj.com/site/about/guidelines.xhtml

Sensitivity analyses

Our pre-planned sensitivity analyses will only be performed for the WISC-V, the Movement-ABC and the CBCL total scores to minimize the effect of multiple testing.

<u>Imputation missing data:</u> A sensitivity analysis using imputation techniques will be performed to impute missing data for children that are lost to follow-up. Imputation techniques will only be applied when it can be assumed that data is (mostly) missing at random and the follow-up rate is follow-up rate ≥70% (the group agreed on an arbitrary). If the loss to follow-up rate is higher a best- and worst-case scenario will be performed. In these two scenarios the missing cases are imputed either all as 'normal' (best case) or as 'abnormal' (worst case) outcomes. These scenarios will provide some insight on the robustness of the complete case follow-up results.

<u>Age and puberty adjusted scores:</u> Despite the fact that most children are born late premature/near term or full term, a sensitivity analyses will be performed using age-adjusted scores (corrected for prematurity). Finally, a sensitivity analysis using results of the PDS indicating child's puberty status will be performed.

Subgroup analyses exploring the potential impact of effect modification

<u>Dealing with the effect of 'down-stream' factors:</u> During time to follow-up (due to loss to follow-up) a substantial difference in the prevalence 'down-stream' factors could potentially appear ('down-stream' factors are defined as potential effect modifiers appearing after randomization, such as sepsis at birth, positive GBS). In sensitivity analysis the potential interaction of the following 'down-stream' factors will be explored: gestational age at PROM, receiving antibiotics, receiving steroids for fetal maturation, receiving tocolysis, group B streptococci (GBS) positivity, a positive vaginal culture (including GBS and other pathogens not consistent with normal flora), neonatal sepsis, and for women who participated in the former follow-up study of children at 2 years of age.(9) The analysis will be stratified by these different factors and the potential differences in long term outcomes between the different strata will be explored.

For peer review only - http://bmjopen.bmj.com/site/about/guidelines.xhtml

A P-value of <0.05 was considered to indicate statistical significance. All analyses will be performed according to the intention-to-treat principle using IBM SPSS (NY, USA) or in RStudio (Boston, MA).

A statistical analysis plan (SAP), reporting a more detailed description of the statistical methods and analyses, will be published separately from the PPROMEXIL follow-up protocol.

Patient and public involvement

The Dutch association for parents of incubator children and the Dutch Collaboration of parent- and patient organisations endorsed the study and provided input on the study proposal. Parents from the Dutch association for parents of incubator children participated in an online survey. Additionally, mothers of prematurely born children participated in a focus group meeting organized by our research team, to discuss the different aspects of child's long-term development to incorporate in long-term follow-up research.

DISCUSSION

Long-term follow-up of all children born to mothers participating in obstetric intervention trials is of crucial importance.(26) The outcome late neurodevelopmental morbidity has been identified and selected by parents as one of 13 core outcomes for studies evaluating preventive interventions for preterm birth.(27) Furthermore, previous studies have stressed the importance of long-term follow-up by demonstrating that interventions performed during pregnancy can have unexpected long-term effects on children which may not be apparent at birth or during neonatal assessment.(28) By assessing cognition, motor function, behaviour, respiratory problems, general health and school attainment in an extensive and structured follow-up, this study will have the unique opportunity to help understanding the long-term

For peer review only - http://bmjopen.bmj.com/site/about/guidelines.xhtml

effects of our current treatment regimen for late preterm PROM on women's offspring. Results from our study should be validated in other follow-up studies comparing induction of labour to expectant management.

For peer review only

For peer review only - http://bmjopen.bmj.com/site/about/guidelines.xhtml

ETHICS AND DISSEMINATION

Ethics approval and consent to participate

The PPROMEXIL Follow-up trial aims to assess long-term childhood outcomes of the PPROMEXIL trial (ISRCTN29313500) and PPROMEXIL-2 trial (MEC 05-240, ISRCTN05689407), two multicentre RCTs using the same study protocol. The Medical Ethics Committee of the Academic Medical Centre Amsterdam (MEC) has approved the PPROMEXIL Follow-up trial (MEC2016_217, NL58494.018.16). Table 1 describes the chronology of submission and amendments to the MEC.

Table 1.

Chronology submission and revisions PPROMEXIL follow-up study

**Version** no.	**Date** (DD-MM-YYYY)	**Main reasons for change**
**1**	29-09-2016	N/A, first submission to MEC
**2**	20-12-2017	MEC2016_217#C20161752<br>Modifications requested by MEC d.d. 05-09-2016
**3**	04-01-2018	Modifications requested by MEC d.d. 04-01-2018<br>Additional information on informed consent about saving data (mother and child) up to 15 years after trial
**3**	10-01-2018	Approval MEC d.d. 10-01-2018
**4**	31-05-2018	Amendment 1:<br>- Administrative modifications<br>- Change of acronym to PPROMEXIL follow-up<br>- Addition of two questionnaires (ISAAC and Puberty developmental scale)
**4**	04-07-2018	Approval amendment version 4 d.d. 04-07-2018
**5**	10-08-2018	Amendment 2:<br>- Modification in protocol on how to recruit participants

For peer review only

For peer review only - http://bmjopen.bmj.com/site/about/guidelines.xhtml

		(via research nurses and PhD student)<br>- Modification in Patient Information Files on recruitment
**5**	23-08-2018	Approval amendment version 5 d.d. 23-08-2018
**6**	06-11-2018	Amendment 3:<br>- Clarification on informed consent procedure, parents and participants will be counseled through the telephone and sign informed consent at home<br>- Minor modifications in the general health questionnaire
**6**	23-11-2018	Approval amendment version 6 d.d. 23-11-2018
**7**	03-04-2019	Amendment 4:<br>- Addition of patient information files for children age 12-15
**7**	12-04-2019	Approval amendment version 7 d.d. 12-04-2019

See https://www.zorgevaluatienederland.nl/evaluations/ppromexil-follow-up for the full study protocol and electronic case record form. Written informed consent will be obtained from both parents prior to the examination. Children ≥12 years of age have to sign their own informed consent, in addition to the informed consent of their parents, at the day of the assessment. A copy of the informed consent form(s) will be given to the parents/child.

Dissemination

No arrangements have been made concerning public disclosure. The trial is registered in the Dutch Trial register (Trial registration number: NTR6953. Date of registration December 28th 2017). An overview of the WHO trial registration data set is described in Table 2.

For peer review only - http://bmjopen.bmj.com/site/about/guidelines.xhtml

546 **Table 2.**

547 **WHO trial registration data set**

Primary Registry and Trial Identifying Number	Trial NL6623 (NTR6953)
Date of Registration in Primary Registry	December 28th, 2017
Secondary Identifying Numbers	n/a
Source(s) of Monetary or Material Support	ZonMW Dutch Healthcare efficacy program
Primary Sponsor	Academical Medical Center (AMC), Amsterdam, The Netherland
Secondary Sponsor(s)	n/a
Contact for Public Queries	Drs. Noor Simons<br><br>Followup.ppromexil@amsterdamumc.nl
Contact for Scientific Queries	Prof. dr. Eva Pajkrt<br><br>e.pajkrt@amsterdamumc.nl
Public Title	PPROMEXIL follow-up
Scientific Title	Child outcomes after induction of labour or expectant management in women with preterm prelabour rupture of membranes between 34-37 weeks of gestation: the PPROMEXIL Follow-up trial, a long-term follow-up study of the randomised controlled trials PPROMEXIL and PPROMEXIL-2.
Countries of Recruitment	The Netherlands
Health Condition(s) or Problem(s) Studied	Late preterm prelabour rupture of membranes (PROM between 34^{+0} and 36^{+6} weeks gestational age). Long-term effects of induction of labour versus expected management.
Intervention(s)	n/a
Key Inclusion and Exclusion Criteria	The PPROMEXIL Follow-up trial will analyse children of mothers with a singleton pregnancy (induction of labour n=359; expectant management n=352). At 10-12 years of (corrected) age all surviving children will be invited for follow-up.
Study Type	Follow-up of a randomized controlled trial
Date of First Enrollment	August 3rd, 2018

For peer review only - http://bmjopen.bmj.com/site/about/guidelines.xhtml

Sample Size	All sample size calculations are with a power of 90%, a two-sided a of 0.05 and ß of 0.20. To be able to detect a clinically relevant difference in mean scores of 0.5 SD in all tests, 86 children per group will be sufficient (total 172 children). This 0.5 SD equals a difference of 7.5 IQ points in the mean score of the WISC-V test (cognitive development), a difference of 1.5 points on the mean total standard scores of the M-ABC-2 (motor skills) and a difference of 5 points on the mean T scores in any of the broadband problem scales of the CBCL (behavioral development) between both groups. Thus, since 172 children comprise 24% of our total, also in case of limited follow up, differences of 0.5 SD can be picked up.
Recruitment Status	Open for patient inclusion
Primary Outcome(s)	Cognitive development (Wechsler Intelligence Scale for Children, WISC-V)<br><br>Motor skills (Movement-ABC-2)<br><br>Behaviour (Child Behavior Checklist, CBCL).
Key Secondary Outcomes	Academic attainment and behavior (Teacher Report Form, TRF)<br><br>Sensory processing (Short Sensory Profile, SSP)<br><br>Respiratory problems (International Study of Asthma and Allergies in Childhood questionnaire, ISAAC questionnaire)<br><br>Pubertal status (Puberty Developmental Scale, PDS)<br><br>Height, weight, bloodpressure<br><br>General health and demographics (questionnaires)
Ethics Review	The Medical Ethics Committee of the Academic Medical Centre Amsterdam (METC) has approved the PPROMEXIL Follow-up trial (METC 2016_217, NL58494.018.16).
Completion date	n/a
Summary Results	n/a
IPD sharing statement	n/a

Trial results will be submitted to a peer-reviewed journal, regardless of the outcome and made open access available in accordance with the Netherlands Organisation for Health Research and Innovation (ZonMW) policy. Results will be incorporated in national guidelines and patient information leaflets. Co-authorship will be based on the international committee

For peer review only

For peer review only - http://bmjopen.bmj.com/site/about/guidelines.xhtml

of medical journal editor's guidelines. Contributors that not fulfil these criteria will be listed as collaborators. The order of authors will be based on scientific input.

Availability of data and materials

The datasets used and/or analysed during the current study will be available from the corresponding author on reasonable request.

FOOTNOTES

Author's contributions

AdR, NS, JvtH, AvWL, CAM, MvW, GJvB, FV, DvdH, ASPvT, BWM, TR, EP are member of the PPROMEXIL Follow-up trial study group and were involved in conception and design of the study. AdR, NS, JvtH drafted the manuscript which follows the SPIRIT checklist for reporting randomised trials. AdR, NS, JvtH, AvWL, CAM, MvW, GJvB, FV, DvdH, ASPvT, TR, BWM, EP discussed and fine-tuned the final design of the study. All authors edited the manuscript and read and approved the final version of the manuscript.

Funding statement

The study group received funding by ZonMW, the Netherlands Organization for Health Research and Development (governmental funding), grant number: 843002826. ZonMW peer reviewed the primary study protocol, they had no other involvement in study design. ZonMw will not have any involvement in data collection, nor in analysis or writing of the manuscript.

Competing interest statement

Dr. Ben Willem Mol is supported by a NHMRC Practitioner Fellowship (GNT1082548). Dr.

For peer review only

For peer review only - http://bmjopen.bmj.com/site/about/guidelines.xhtml

Ben Willem Mol reports consultancy for ObsEva, Merck Merck KGaA and Guerbet. All other authors did not report any conflicts of interest.

Acknowledgements

We would like to thank the research nurses and research midwives from the Dutch Consortium for Healthcare Evaluation and Research in Obstetrics and Gynecology - NVOG Consortium 2.0 as well as the Dutch Consortium Trialbureau for their efforts. The authors thank the contributors of PPROMEXIL and PPROMEXIL-2 trial. For a list of contributors to the PPROMEXIL and PPROMEXIL-2 trials see Additional file 6.

List of abbreviations

CBCL; Child Behavior Checklist, CI; confidence interval, CWIT; Color-Word Interference Test, DCD; Developmental Coordination Disorder, FSIQ; Full Scale IQ score, GA; gestational age, GBS; group B streptococci, IPDMA; Individual Participant Data Meta-analysis, IQ; intelligence quotient, ISAAC questionnaire; International Study of Asthma and Allergies in Childhood questionnaire, ISRCTN; International Standard Randomised Controlled Trial Number, M-ABC-2; Movement Assessment Battery for Children-2, M.D.; Doctor of Medicine, MEC; Medical ethics committee; in Dutch: medisch ethische toetsingscommissie (METC), NTR; Trial registration number, NVOG; the Dutch college of Obstetricians and Gynecologists, in Dutch: Nederlandse Vereniging voor Gynaecologie en Obstetrie (NVOG), PDS; Puberty Developmental Scale, PROM; prelabour rupture of membranes, PPROMEXIL trial; Preterm Prelabour Rupture Of Membranes EXpectant management vs Induction of Labour trial, PPROMT; the Preterm Pre-labour Rupture of Membranes close to Term Trial, RCT; randomised controlled trial, RR; relative risk, SD; standard deviation, SSP-NL; Short Sensory Profile, TRF; Teacher Report Form, WISC-V-NL; Wechsler Intelligence Scale for Children-V.

For peer review only

Figures and Tables

Figure 1. Overview of PPROMEXIL follow-up participants

Table 1. Chronology submission and revisions PPROMEXIL follow-up study

Table 2. WHO trial registration data set

Additional file 1. SPIRIT checklist for reporting randomised trials

Additional file 2. SPIRIT Schematic diagram of enrolment PPROMEXIL follow-up participants.

Additional file 3. GRIPP2 short checklist PPROMEXIL follow-up

Additional file 4. Patient information PPROMEXIL Follow-up (English version)

Additional file 5. Direct Acyclic Graph (DAG) figure

Additional file 6. Authors PPROMEXIL randomised controlled trials.

For peer review only

REFERENCES

1. index.php?pagina=/richtlijn/item/pagina.php&richtlijn_id= NVvOeGRomboolAhn-dn.

2. van der Ham DP, van der Heyden JL, Opmeer BC, Mulder AL, Moonen RM, van Beek JH, et al. Management of late-preterm premature rupture of membranes: the PPROMEXIL-2 trial. Am J Obstet Gynecol. 2012;207(4):276 e1-10.

3. van der Ham DP, Vijgen SM, Nijhuis JG, van Beek JJ, Opmeer BC, Mulder AL, et al. Induction of labor versus expectant management in women with preterm prelabor rupture of membranes between 34 and 37 weeks: a randomized controlled trial. PLoS Med. 2012;9(4):e1001208.

4. Morris JM, Roberts CL, Bowen JR, Patterson JA, Bond DM, Algert CS, et al. Immediate delivery compared with expectant management after preterm pre-labour rupture of the membranes close to term (PPROMT trial): a randomised controlled trial. Lancet. 2016;387(10017):444-52.

5. Quist-Nelson J, de Ruigh AA, Seidler AL, van der Ham DP, Willekes C, Berghella V, et al. Immediate Delivery Compared With Expectant Management in Late Preterm Prelabor Rupture of Membranes: An Individual Participant Data Meta-analysis. Obstet Gynecol. 2018;131(2):269-79.

6. Vijgen SM, van der Ham DP, Bijlenga D, van Beek JJ, Bloemenkamp KW, Kwee A, et al. Economic analysis comparing induction of labor and expectant management in women with preterm prelabor rupture of membranes between 34 and 37 weeks (PPROMEXIL trial). Acta Obstet Gynecol Scand. 2014;93(4):374-81.

7. Prelabor Rupture of Membranes: ACOG Practice Bulletin, Number 217. Obstet Gynecol. 2020;135(3):e80-e97.

8. 2010. RCoOaGPpromG-tgNLURCoOaG.

9. van der Heyden JL, Willekes C, van Baar AL, van Wassenaer-Leemhuis AG, Pajkrt E, Oudijk MA, et al. Behavioural and neurodevelopmental outcome of 2-year-old children after preterm premature rupture of membranes: follow-up of a randomised clinical trial comparing induction of labour and expectant management. Eur J Obstet Gynecol Reprod Biol. 2015;194:17-23.

10. Armstrong-Wells J, Donnelly M, Post MD, Manco-Johnson MJ, Winn VD, Sebire G. Inflammatory predictors of neurologic disability after preterm premature rupture of membranes. Am J Obstet Gynecol. 2015;212(2):212 e1-9.

11. Allotey J, Zamora J, Cheong-See F, Kalidindi M, Arroyo-Manzano D, Asztalos E, et al. Cognitive, motor, behavioural and academic performances of children born preterm: a meta-analysis and systematic review involving 64 061 children. BJOG: An International Journal of Obstetrics and Gynaecology. 2018;125(1):16-25.

12. Chan AW, Tetzlaff JM, Gotzsche PC, Altman DG, Mann H, Berlin JA, et al. SPIRIT 2013 explanation and elaboration: guidance for protocols of clinical trials. BMJ. 2013;346:e7586.

13. 2019 hwccf-rAoN.

14. Wechsler D. Wechsler Intelligence Scale for Children veNbAPBBV.

15. Henderson SE SDMABfCMLPC.

16. Verhulst VdE, Koot. Handleiding voor de CBCL/4-18. Rotterdam: Sophia Kinderziekenhuis, Erasmus MC. 1996. .

17. Verhulst FC VdEJHvdTsRFTRAK-eJ, Sophia Kinderziekenhuis / Erasmus MC. 2000.

18. Rietman A. SP-NHPAaI, Amsterdam. 2013.

19. Asher MI, Keil U, Anderson HR, Beasley R, Crane J, Martinez F, et al. International Study of Asthma and Allergies in Childhood (ISAAC): rationale and methods. Eur Respir J. 1995;8(3):483-91.

20. Sole D, Camelo-Nunes IC, Wandalsen GF, Mallozi MC. Asthma in children and adolescents in Brazil: contribution of the International Study of Asthma and Allergies in Childhood (ISAAC). Rev Paul Pediatr. 2014;32(1):114-25.

21. Carskadon MA, Acebo C. A self-administered rating scale for pubertal development. J Adolesc Health. 1993;14(3):190-5.

22. Simons NE, van de Beek C, van der Lee JH, Opmeer BC, van Wassenaer-Leemhuis AG, van Baar AL, et al. Child outcomes after placement of a cervical pessary in women with a multiple

For peer review only

For peer review only - http://bmjopen.bmj.com/site/about/guidelines.xhtml

pregnancy: A 4-year follow-up of the ProTWIN trial. Acta Obstet Gynecol Scand. 2019;98(10):1292-300.
23. van 't Hooft J, van der Lee JH, Opmeer BC, van Wassenaer-Leemhuis AG, van Baar AL, Bekedam DJ, et al. Pessary for prevention of preterm birth in twin pregnancy with short cervix: 3-year follow-up study. Ultrasound Obstet Gynecol. 2018;51(5):621-8.
24. van Os MA, van der Ven AJ, Kleinrouweler CE, Schuit E, Kazemier BM, Verhoeven CJ, et al. Preventing Preterm Birth with Progesterone in Women with a Short Cervical Length from a Low-Risk Population: A Multicenter Double-Blind Placebo-Controlled Randomized Trial. Am J Perinatol. 2015;32(10):993-1000.
25. Hughes RA, Heron J, Sterne JAC, Tilling K. Accounting for missing data in statistical analyses: multiple imputation is not always the answer. Int J Epidemiol. 2019;48(4):1294-304.
26. Teune MJ, van Wassenaer AG, Malin GL, Asztalos E, Alfirevic Z, Mol BW, et al. Long-term child follow-up after large obstetric randomised controlled trials for the evaluation of perinatal interventions: a systematic review of the literature. BJOG. 2013;120(1):15-22.
27. van 't Hooft J, Duffy JM, Daly M, Williamson PR, Meher S, Thom E, et al. A Core Outcome Set for Evaluation of Interventions to Prevent Preterm Birth. Obstet Gynecol. 2016;127(1):49-58.
28. Kenyon S, Pike K, Jones DR, Brocklehurst P, Marlow N, Salt A, et al. Childhood outcomes after prescription of antibiotics to pregnant women with spontaneous preterm labour: 7-year follow-up of the ORACLE II trial. Lancet. 2008;372(9646):1319-27.

For peer review only

For peer review only - http://bmjopen.bmj.com/site/about/guidelines.xhtml

Figure 1. Overview of PPROMEXIL follow-up participants

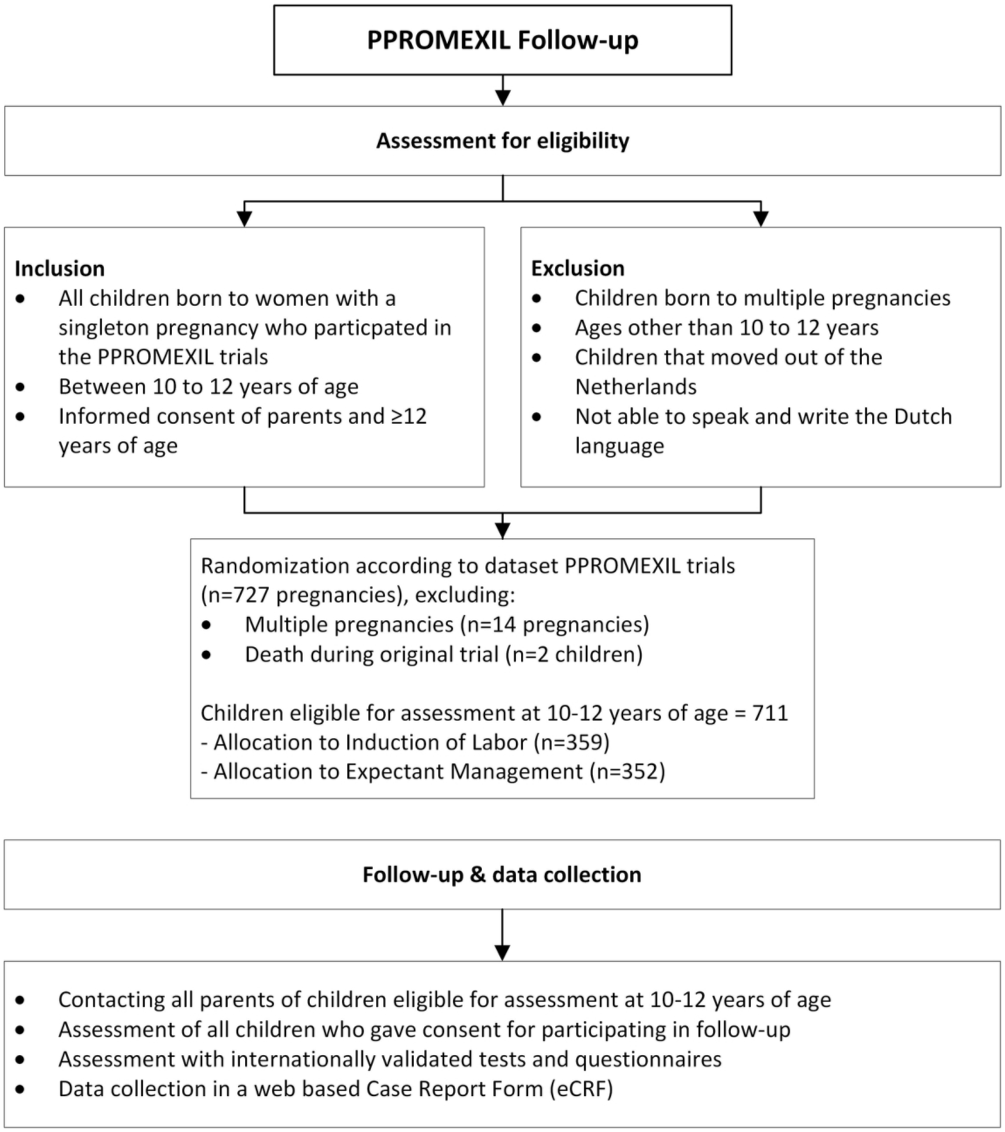

Figure 1. Overview of PPROMEXIL follow-up participants

144x170mm (300 x 300 DPI)

Additional file 1. SPIRIT checklist for reporting randomized trials

Section/item	Item No	Description	Adressd on page number
**Administrative information**			
Title	1	Descriptive title identifying the study design, population, interventions, and, if applicable, trial acronym	1
Trial registration	2a	Trial identifier and registry name. If not yet registered, name of intended registry	5, 10, 25-26
	2b	All items from the World Health Organization Trial Registration Data Set	5, 10, 25-26 Table 2
Protocol version	3	Date and version identifier	NA
Funding	4	Sources and types of financial, material, and other support	5, 25-27
Roles and responsibilities	5a	Names, affiliations, and roles of protocol contributors	1-3, 27
	5b	Name and contact information for the trial sponsor	3
	5c	Role of study sponsor and funders, if any, in study design; collection, management, analysis, and interpretation of data; writing of the report; and the decision to submit the report for publication, including whether they will have ultimate authority over any of these activities	27
	5d	Composition, roles, and responsibilities of the coordinating centre, steering committee, endpoint adjudication committee, data management team, and other individuals or groups overseeing the trial, if applicable (see Item 21a for data monitoring committee)	NA

For peer review only

Introduction

Background and rationale	6a	Description of research question and justification for undertaking the trial, including summary of relevant studies (published and unpublished) examining benefits and harms for each intervention	7-9
	6b	Explanation for choice of comparators	7-9
Objectives	7	Specific objectives or hypotheses	8, 9
Trial design	8	Description of trial design including type of trial (eg, parallel group, crossover, factorial, single group), allocation ratio, and framework (eg, superiority, equivalence, noninferiority, exploratory)	10, 11

Methods: Participants, interventions, and outcomes

Study setting	9	Description of study settings (eg, community clinic, academic hospital) and list of countries where data will be collected. Reference to where list of study sites can be obtained	10-12
Eligibility criteria	10	Inclusion and exclusion criteria for participants. If applicable, eligibility criteria for study centres and individuals who will perform the interventions (eg, surgeons, psychotherapists)	10-12
Interventions	11a	Interventions for each group with sufficient detail to allow replication, including how and when they will be administered	12-17
	11b	Criteria for discontinuing or modifying allocated interventions for a given trial participant (eg, drug dose change in response to harms, participant request, or improving/worsening disease)	11
	11c	Strategies to improve adherence to intervention protocols, and any procedures for monitoring adherence (eg, drug tablet return, laboratory tests)	NA
	11d	Relevant concomitant care and interventions that are permitted or prohibited during the trial	NA

For peer review

Outcomes	12	Primary, secondary, and other outcomes, including the specific measurement variable (eg, systolic blood pressure), analysis metric (eg, change from baseline, final value, time to event), method of aggregation (eg, median, proportion), and time point for each outcome. Explanation of the clinical relevance of chosen efficacy and harm outcomes is strongly recommended	13-17
Participant timeline	13	Time schedule of enrolment, interventions (including any run-ins and washouts), assessments, and visits for participants. A schematic diagram is highly recommended	10, 17, Figure1, Additional file 2
Sample size	14	Estimated number of participants needed to achieve study objectives and how it was determined, including clinical and statistical assumptions supporting any sample size calculations	18, 19
Recruitment	15	Strategies for achieving adequate participant enrolment to reach target sample size	11, 12
**Methods: Assignment of interventions (for controlled trials)**			
Allocation:			
Sequence generation	16a	Method of generating the allocation sequence (eg, computer-generated random numbers), and list of any factors for stratification. To reduce predictability of a random sequence, details of any planned restriction (eg, blocking) should be provided in a separate document that is unavailable to those who enrol participants or assign interventions	10-12
Allocation concealment mechanism	16b	Mechanism of implementing the allocation sequence (eg, central telephone; sequentially numbered, opaque, sealed envelopes), describing any steps to conceal the sequence until interventions are assigned	10-12
Implementation	16c	Who will generate the allocation sequence, who will enrol participants, and who will assign participants to interventions	10-12

Blinding (masking)	17a	Who will be blinded after assignment to interventions (eg, trial participants, care providers, outcome assessors, data analysts), and how	11-12
	17b	If blinded, circumstances under which unblinding is permissible, and procedure for revealing a participant's allocated intervention during the trial	NA
**Methods: Data collection, management, and analysis**			
Data collection methods	18a	Plans for assessment and collection of outcome, baseline, and other trial data, including any related processes to promote data quality (eg, duplicate measurements, training of assessors) and a description of study instruments (eg, questionnaires, laboratory tests) along with their reliability and validity, if known. Reference to where data collection forms can be found, if not in the protocol	12-17, 24
	18b	Plans to promote participant retention and complete follow-up, including list of any outcome data to be collected for participants who discontinue or deviate from intervention protocols	NA
Data management	19	Plans for data entry, coding, security, and storage, including any related processes to promote data quality (eg, double data entry; range checks for data values). Reference to where details of data management procedures can be found, if not in the protocol	12
Statistical methods	20a	Statistical methods for analysing primary and secondary outcomes. Reference to where other details of the statistical analysis plan can be found, if not in the protocol	18-21
	20b	Methods for any additional analyses (eg, subgroup and adjusted analyses)	20, 21
	20c	Definition of analysis population relating to protocol non-adherence (eg, as randomised analysis), and any statistical methods to handle missing data (eg, multiple imputation)	20, 21

Methods: Monitoring

Data monitoring	21a	Composition of data monitoring committee (DMC); summary of its role and reporting structure; statement of whether it is independent from the sponsor and competing interests; and reference to where further details about its charter can be found, if not in the protocol. Alternatively, an explanation of why a DMC is not needed	12
	21b	Description of any interim analyses and stopping guidelines, including who will have access to these interim results and make the final decision to terminate the trial	NA
Harms	22	Plans for collecting, assessing, reporting, and managing solicited and spontaneously reported adverse events and other unintended effects of trial interventions or trial conduct	NA
Auditing	23	Frequency and procedures for auditing trial conduct, if any, and whether the process will be independent from investigators and the sponsor	NA

Ethics and dissemination

Research ethics approval	24	Plans for seeking research ethics committee/institutional review board (REC/IRB) approval	10, 23-25
Protocol amendments	25	Plans for communicating important protocol modifications (eg, changes to eligibility criteria, outcomes, analyses) to relevant parties (eg, investigators, REC/IRBs, trial participants, trial registries, journals, regulators)	NA
Consent or assent	26a	Who will obtain informed consent or assent from potential trial participants or authorised surrogates, and how (see Item 32)	11, 24
	26b	Additional consent provisions for collection and use of participant data and biological specimens in ancillary studies, if applicable	NA

For peer review only - http://bmjopen.bmj.com/site/about/guidelines.xhtml

Confidentiality	27	How personal information about potential and enrolled participants will be collected, shared, and maintained in order to protect confidentiality before, during, and after the trial	12
Declaration of interests	28	Financial and other competing interests for principal investigators for the overall trial and each study site	24-28
Access to data	29	Statement of who will have access to the final trial dataset, and disclosure of contractual agreements that limit such access for investigators	27
Ancillary and post-trial care	30	Provisions, if any, for ancillary and post-trial care, and for compensation to those who suffer harm from trial participation	NA
Dissemination policy	31a	Plans for investigators and sponsor to communicate trial results to participants, healthcare professionals, the public, and other relevant groups (eg, via publication, reporting in results databases, or other data sharing arrangements), including any publication restrictions	11, 26, 27
	31b	Authorship eligibility guidelines and any intended use of professional writers	27
	31c	Plans, if any, for granting public access to the full protocol, participant-level dataset, and statistical code	27
**Appendices**			
Informed consent materials	32	Model consent form and other related documentation given to participants and authorised surrogates	10, 11, 23, 29 additional file 4
Biological specimens	33	Plans for collection, laboratory evaluation, and storage of biological specimens for genetic or molecular analysis in the current trial and for future use in ancillary studies, if applicable	NA

*It is strongly recommended that this checklist be read in conjunction with the SPIRIT 2013 Explanation & Elaboration for important clarification on the items. Amendments to the protocol should be tracked and dated. The SPIRIT checklist is copyrighted by the SPIRIT Group under the Creative Commons "Attribution-NonCommercial-NoDerivs 3.0 Unported" license

For peer review only

Additional file 2. SPIRIT schematic diagram of schedule of enrolment, interventions, and assessments of the women participating in PPROMEXIL trials and children participating in PPROMEXIL follow-up

	STUDY PERIOD Original PPROMEXIL trials - women			STUDY PERIOD PPROMEXIL Follow-up - children		
	**Enrolment original trials**	**Allocation original trials**	**Outcomes original trials**	**Enrolment follow-up study**	**Assessment follow-up study**	**Close-out**
**TIMEPOINT**	*Women with PPROM between 34 and 36+6 weeks of gestation*	t = 0	*Pregnancy, childbirth and neonatal period*	*After 10 – 12 years*	*Age 10-12 years*	*Age 10-12 years*
**ENROLMENT:**						
**Eligibility screen**	X			X		
**Informed consent**	X			X	X	
**Allocation**		X				
**INTERVENTIONS:**						
**Induction of Labor**		X				
**Expectant Management**		X				
**ASSESSMENTS:**						
Baseline variables	X	X		X		
Outcome variables			X		X	X

For peer review

Additional file 3. GRIPP2 short form

Section and topic	Item	Reported on page No
1: Aim	Report the aim of PPI in the study	8, 20-21
2: Methods	Provide a clear description of the methods used for PPI in the study	8, 20-21
3: Study results	Outcomes—Report the results of PPI in the study, including both positive and negative outcomes	8
4: Discussion and conclusions	Outcomes—Comment on the extent to which PPI influenced the study overall. Describe positive and negative effects	NA
5: Reflections/critical perspective	Comment critically on the study, reflecting on the things that went well and those that did not, so others can learn from this experience	NA

For peer review only

Additional file 4. Patient information PPROMEXIL Follow-up (English version)

Patient information
PPROMEXIL Follow-up trial

Child outcomes after induction of labour or expectant management in women with preterm prelabour rupture of membranes between 34-37 weeks of gestation: the PPROMEXIL Follow-up trial, a long-term follow-up study of the randomised controlled trials PPROMEXIL and PPROMEXIL-2.

Dear Sir/Madame,

We would like to inform you on this research project called: 'Child outcomes after induction of labour or expectant management in women with preterm prelabour rupture of membranes between 34-37 weeks of gestation: the PPROMEXIL Follow-up trial, a long-term follow-up study of the randomised controlled trials PPROMEXIL and PPROMEXIL-2.'. You and your child have been asked to to take part in a medical-scientific study. Participation requires your written consent. In this letter we would like to inform you on the purpose of this research project and any advantages or disadvantages that it may hold for you. Please read this information carefully and do not hesitate to ask the investigator for an explanation if you have any questions. You can also ask the independent expert, who is mentioned at the end of this document, for additional information regarding the study protocol (page 4). And you may also discuss it with your partner, friends or family. Additional (general) information about participating in a study can be found in the enclosed general brochure on medical research.

Introduction
In the past you have participated in a trial called: PPROMEXIL or PPROMEXIL-2. You have participated in this trial because during your (last) pregnancy you have been diagnosed with premature preterm rupture of the fetal membranes (PPROM) at 34-37 weeks' gestational age. You have been treated with either expectant management or induction of labor. Short-term outcomes of your pregnancy and your child have been assessed at that time. In the proposed follow-up trial we would like to investigate offspring's long-term outcomes of women who participated in the PPROMEXIL trial; such as offspring's cognitive- and neurodevelopment, motor skills, behavioral development and general health.

Purpose of the research protocol
The goal of this study is to assess the long-term effects on children born to mothers whose pregnancy was complicated by PPROM between 34-37 weeks and who were treated with either induction of labor or expected management (PPROMEXIL and PPROMEXIL-2 trial). We would like to investigate child's cognitive development (intelligence), neurodevelopment

For peer review only - http://bmjopen.bmj.com/site/about/guidelines.xhtml

(fine and gross motor skills), academic attainment (school results), behavioral development and general health (diseases, hospital admissions, respiratory problems, but also length, weight, growth).

What participation involves

If you decide to participate in our research protocol we will contact you by phone. All data will be collected during one visit in a local or academic hospital in the neighborhood. During this visit children will be assessed on cognitive- and neurodevelopment and general health. Also physical examination will be obtained. Assessment of children has a playful approach, is enjoyable for most children and is not invasive. Furthermore, we will ask you to fill out questionnaires. Filling out these questionnaires will cost approximately 35 minutes. If necessary we would like to ask your permission to look up details on your (last) pregnancy and delivery in your medical chart. Furthermore, we would like to ask permission to obtain data from your child's consultation bureau or general practitioner.

Also, we would like to assess your child's academic attainment and school performance. Therefore we would like to ask the teacher at school to fill out a short questionnaire. We will ask the teacher whether your child needs any special education or additional support teaching.

If appreciated you can receive a short report on your child's cognitive, motor and behaviour development as measured by the different neurodevelopmental tests (WISC-V-NL, M-ABC-2, and CBCL). This short report will give information on total test scores and will tell you whether the test results of your child are above, on or below average. If the test results are below average, we will be advise you to contact your general practioner or a psychologist (in Dutch: 'GZ psycholoog of klinisch praktiserend psycholoog') for further help and interpretation of the different test scores.

For peer review only - http://bmjopen.bmj.com/site/about/guidelines.xhtml

Summary of study protocol

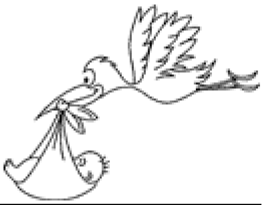

During your (last) pregnancy you have participated in the PPROMEXIL trial

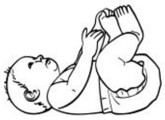

Short term outcomes of your child have been assessed shortly after birth

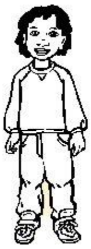

Years passed by and we would like to know how your son or daughter is developing. How is he/she doing at school? And is he/she good at sports?

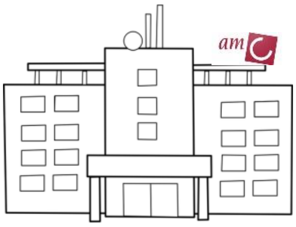

We would like to investigate your son or daughter in a local hospital in your neighborhood during a single visit

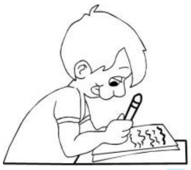

Together we will make some tests. Some of these tests may be easy, while others could be a little bit more difficult

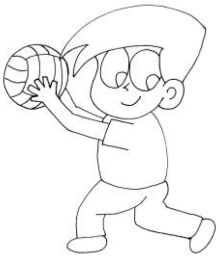

Also, we would like to investigate whether he or she is good at sports. And a physical examination will be obtained.

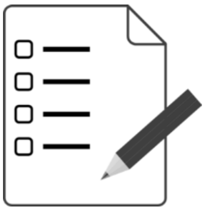

We would like to ask you to fill out four questionnaires about your son or daughter

For peer review only

For peer review only - http://bmjopen.bmj.com/site/about/guidelines.xhtml

Possible advantages and disadvantages

If you and your child participate in this research project it will cost time. Assessment of children has a playful approach, is enjoyable for most children and is not invasive. Participation in this trial is not associated with any risks.

You will not experience any personal benefit from participation in this study. However, your participation may contribute to more knowledge on the long-term (treatment) effects of induction of labour or expectant management on women's offspring after preterm prelabor rupture of the fetal membranes (PPROM) at a gestational age of 34-37 weeks.

Voluntary participation

It is up to you to decide whether or not you and your child will participate in the study. Participation is voluntary. If you do participate in the study, you can always change your mind and decide to stop, at any time during this study, without stating a reason. The data collected until that time will still be used for the study.
Your participation in this research will not change any decision making and quality of care that would be normally given to you or your child.

Confidential information

We assure that all data collected during the study will remain confidential. Data will be obtained in a coded manner. The investigator is the only person who will know which code you have. The key to the code will stay with the investigator. In the reports about the study only use this code will be used.You will not find your name in scientific papers.

Some people may access your medical and personal data. This is to check whether the study has been conducted in a good and reliable manner. General information about this policy can be found in the general brochure on medical research. People who may access your and your child's data are: the study team, a monitor of the study and the Healthcare Inspectorate. They will keep the data a secret. If you sign the consent form, you consent to your medical and personal data being collected, stored and accessed. The investigator will store your data for 15 years.

Study subject insurance

This study is not associated with any risks for you or your child. Therefore, the Academic Medical Center (AMC) does not need to take out additional insurance.

Compensation for participation

You and your child will not be paid for your participation in this study. You will be reimbursed for your travel costs.

Signing the consent form

When you have had sufficient time for reflection, you will be asked to decide on participation in this study. If you give permission, we will ask you to confirm this in writing on the appended consent form. By your written permission you indicate that you have understood the information and consent to participation in the study. The signature sheet is kept by your attending physician. You will get a copy or a second copy of this consent form.

For peer review only - http://bmjopen.bmj.com/site/about/guidelines.xhtml

Finally

If you have any questions, please contact the research team. You can contact one of the investigators (Noor Simons, PhD student, number; 020 – 5661470) or with the principal investigator in the AMC (Prof. dr. E. Pajkrt, gynecologist-perinatologist, number: 020 – 5661279). If you would like any independent advice about participation in this study, you may contact Dr. J.W. Ganzevoort, gynaecologist. He knows about the study but is not involved in it. Contact details: Dr. J.W. Ganzevoort, gynaecologist, department of Obstetrics and Gynaecology, Academic Medical Center (tel: 020-56 63769).

Thank you for your attention.

Kind Regards,

Prof. dr. E. Pajkrt,
Principal investigator
Professor Fetal and Maternal Medicine
Department of Gynecology and Obstetrics
Academic Medical Center, Amsterdam

Prof. dr. T. Roseboom
Professor of Early Development and Health
Department of Clinical Epidemiology, Biostatistics and Bioinformatics
Department of Gynecology and Obstetrics
Academic Medical Center, Amsterdam

For peer review only

For peer review only - http://bmjopen.bmj.com/site/about/guidelines.xhtml

Informed consent PPROMEXIL Follow-up - parents

- ✓ I have read the information sheet. I was also able to ask questions. My questions have been answered to my satisfaction. I have had enough time to decide whether me and my child will participate in this study.
- ✓ I know that participation is voluntary. I know that me and my child may decide at any time not to participate after all or to withdraw from the study.
- ✓ I know that some people will be able to access this person's personal data. These people are listed in this information sheet.
- ✓ I give permission for information to be requested from my gynaecologist about my pregnancy and delivery.
- ✓ I give permission to fill out questionnaires about my child, and I agree with one physical exam and one neurodevelopmental exam with my child (WISC-V and M-ABC-2).
- ✓ I give permission for information (regarding myself or my child) to be requested from my general practitioner or the GGD ('consultatiebureau').
- ✓ I know that it's possible to be contacted in the future (by post or telephone) after this study for another follow-up research project, if I give consent for this.
- ✓ I consent to my data and data regarding my child being stored at the research location for another 15 years after this study.

I agree to participation in this study

Date:____ - ____ - _________

First and last name child:: ……………………………………………………………………………………….

Child's date of birth: ……………………………………………………………………………………

First and last name child's parent/guardian: …………………………………………………………...

Signature child's parent/guardian:

First and last name child's second parent/guardian: ……………………………………………………

☐ Not applicable, because:……………………………………………………………………......

Signature child's second parent/guardian:

For peer review only

Additional:

I give consent for being contacted again (by post or by telephone) after this study for a follow-up study

☐ I do ☐ I do not

To be signed by the AMC investigator:

I declare that I have fully informed this/these person(s) about this study.

Name of investigator (or his/her representative):…………………………………………………………………

Date:____ - ____ - _________

Signature:

The study subject will receive the full information sheet, together with a copy of the signed consent form.

For peer review only

For peer review only - http://bmjopen.bmj.com/site/about/guidelines.xhtml

Informed consent PPROMEXIL Follow-up – child 12 year and older

- I have read the information sheet. I was also able to ask questions. My questions have been answered to my satisfaction. I have had enough time to decide whether I will participate in this study.
- I know that participation is voluntary. I know that me and my child may decide at any time not to participate after all or to withdraw from the study.
- I know that some people will be able to access this person's personal data. These people are listed in this information sheet.
- I give permission to fill out one questionnaire, and I agree with one physical exam and one neurodevelopmental exam (WISC-V and M-ABC-2).
- I know that it's possible to be contacted in the future (by post or telephone) after this study for another follow-up research project, if I give consent for this.
- I consent to my data being stored at the research location for another 15 years after this study.

I have read all above and the information letter and want to participate with this research.

Date:____ - ____ - _________

First and last name child:……………………………………………………………………………………

Date of birth child: ……………………………………………………………………………………

Signature child: ……………………………………………………………………………………

My parent(s)/guardian(s) also agree with participation in this study and sign informed consent on a separate form.

For peer review only - http://bmjopen.bmj.com/site/about/guidelines.xhtml

Additional file 5. Direct Acyclic Graph (DAG) identifying potential confounding measures

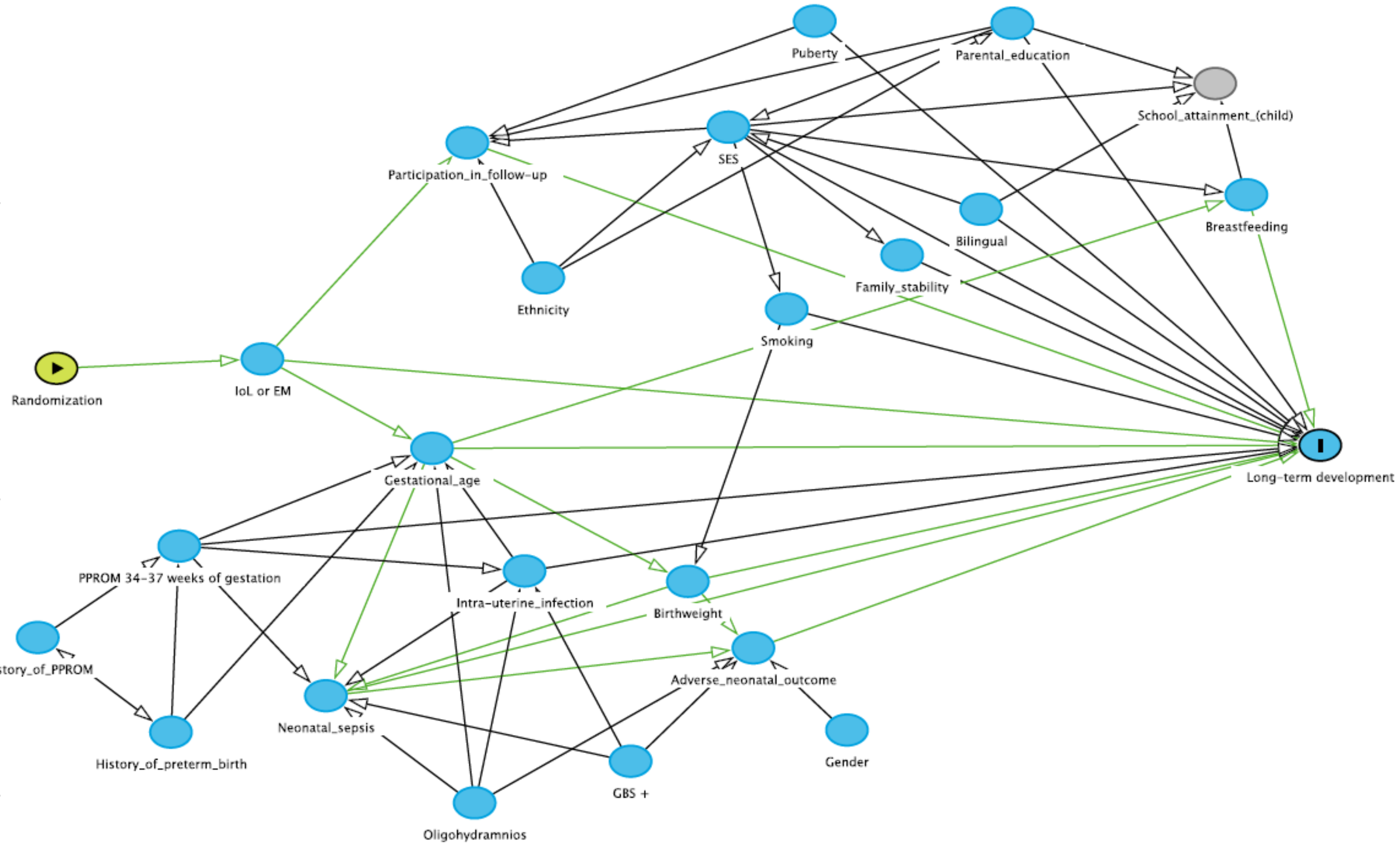

w only

Additional file 6.

Contributors to the PPROMEXIL and PPROMEXIL-2 trials

David P. van der Ham, Christine Willekes, Jantien L. van der Heyden, Sylvia M. C. Vijgen, Jan G. Nijhuis, Johannes J. van Beek, Brent C. Opmeer, Antonius L. M. Mulder, Rob Moonen, Mariët Groenewout, Mariëlle G. van Pampus, Gerald D. Mantel, Kitty W. M. Bloemenkamp, Wim J. van Wijngaarden, Marko Sikkema, Monique C. Haak, Paula J. M. Pernet, Martina Porath, Jan F. M. Molkenboer, Simone Kuppens, Anneke Kwee, Michael E. Kars, Mallory Woiski, Martin J. N. Weinans, Hajo I. J. Wildschut, Bettina M. C. Akerboom, Maureen T.M. Franssen, Christianne J.M. de Groot, J. Hans J. Duvekot, Bettina M.C. Akerboom, Aren J. van Loon, Jan W. de Leeuw, Ben Willem Mol, Aleid G. Leemhuis, E. Pajkrt, Martijn A. Oudijk, Bas Nij Bijvank, Caroline J. Bax, Janneke van ‘t Hooft.

For peer review only
